# Tackling exclusion: A pilot mixed method quasi-experimental identity capital intervention for young people exiting homelessness

**DOI:** 10.1371/journal.pone.0256288

**Published:** 2021-08-20

**Authors:** Naomi S. Thulien, Andrea Wang, Caitlin Mathewson, Ri Wang, Stephen W. Hwang

**Affiliations:** 1 MAP Centre for Urban Health Solutions, Li Ka Shing Knowledge Institute of St Michael’s Hospital, Toronto, Ontario, Canada; 2 Dalla Lana School of Public Health, University of Toronto, Toronto, Ontario, Canada; 3 Centre for Critical Qualitative Health Research, University of Toronto, Toronto, Ontario, Canada; 4 School of Medicine, Boston University, Boston, Massachusetts, United States of America; 5 School of Nursing, McMaster University, Hamilton, Ontario, Canada; 6 Division of General Internal Medicine, Department of Medicine, University of Toronto, Toronto, Ontario, Canada; Chapin Hall at the University of Chicago, UNITED STATES

## Abstract

**Background:**

Longitudinal studies examining the life trajectories of young people after they have exited homelessness have identified concerns with persistent social and economic exclusion, struggles to shake off identities of homelessness, and housing instability. This pilot study sought to explore the feasibility of improving socioeconomic inclusion outcomes by bolstering identity capital (sense of purpose and control, self-efficacy and self-esteem) among young people who had experienced homelessness.

**Methods:**

Nineteen individuals (aged 18–26) who had transitioned out of homelessness within the past three years participated in a six-week, six-session program focused on building identity capital. The study employed a mixed method prospective cohort hybrid design with an intervention group (Group One) and a delayed intervention comparison group (Group Two). Participants were interviewed every three months until nine months post-intervention.

**Results:**

None of the youth who began the intervention dropped out of the program, with the exception of one participant who moved across the country and was unable to continue. Immediately after participating in the intervention, Group One had statistically significant improvements (*p* < .05) and large to very large effect sizes in self-esteem (*d* = 1.16) and physical community integration (*d* = 1.79) compared to changes in Group Two over the same period, which had not yet begun the intervention. In the pooled analysis, small to moderate effect sizes in hopelessness, physical community integration, and self-esteem were observed at all post-intervention time points. Notably, at six- and nine-months post-intervention, statistically significant improvements (*p* < .05) and moderate effect sizes in hopelessness (*d* = -0.73 and *d* = -0.60 respectively) and self-esteem (*d* = 0.71 and *d* = 0.53 respectively) were observed. Youth shared they appreciated the normalizing (vs. pathologizing) of strategies they needed to learn and spoke of the importance of framing new skills as something one needs “to have a better life” vs. “to get better.”

**Conclusions:**

These early findings signal that targeting identity capital is feasible and may be a promising approach to incorporate into a more complex intervention that includes housing, education, and employment supports to help youth transition out of homelessness. Future research could build on these findings through a sufficiently powered randomized controlled trial.

## Introduction

A great deal is known about the social structural inequities associated with young people entering and remaining entrenched in homelessness (e.g., intergenerational poverty, childhood abuse, inadequate education, and limited employment opportunities), but much less about how to sustain transitions off the streets and facilitate meaningful socioeconomic inclusion [1–4]. Findings from the handful of longitudinal studies examining the life-trajectories of young people after they have become “successfully housed” are concerning–most remain socially and economically excluded, struggle to shake off identities of homelessness, and just one misstep away from returning to the streets [3–5]. Moreover, evidence-based solutions designed with the primary aim of addressing inclusion-related challenges among this population are scarce [6–10].

The notion of “ending homelessness” is often linked to the provision of stable housing–the idea being that the ontological security associated with being housed will lead to meaningful socioeconomic inclusion. However, the largest randomized controlled trial to date of Housing First–rent subsidies and recovery-oriented case management supports with no preconditions–was able to demonstrate housing stability, but unable to significantly impact inclusion-related outcomes such as community functioning (e.g., sense of belonging) and quality of life (e.g., sense of well-being) among the subset of youth participants (average age 22 years) two years after randomization to the intervention [11] or among the subset of Toronto, Canada participants (average age 40 years) six years after randomization to the intervention [12] relative to treatment as usual. While housing stability is clearly important, there have been growing calls to move discourse beyond a primary focus on housing stability and critically examine–alongside the voices of those with lived expertise–how to intervene on outcomes associated with meaningful societal inclusion [6, 13, 14]. Key principles of the recovery-oriented approach embedded in Housing First include fostering a sense of hope, focusing on strengths, affirming self-determination, supporting social inclusion and advocacy on the social determinants of health, respect for diversity (e.g., racial and gender identity), and workforce development and planning [15]. In keeping with these concerns, the authors of the aforementioned Housing First study with six-year outcome data, suggest a more targeted recovery-oriented approach may be warranted [12].

Identity capital has been conceptualized as a key determinant of socioeconomic inclusion for young people exiting homelessness [16]. Identity capital refers to the set of internal resources (capital) that people draw on to push forward when life becomes challenging [17]. These identity-based resources include a sense of purpose and control, along with self-efficacy and self-esteem, and are shaped through socially constructed messaging. In other words, our notions about who we are and what we are capable of becoming are formed by the explicit (what people say) and implicit (where we live, our social class, race, gender, etc.) messages we receive. Importantly, we all tend to act in ways that align with our identities. Thus, when faced with adversity, those with low identity capital are more likely to give up and take the path of least resistance–a sort of learned helplessness–while those with high identity capital tend to persevere in the belief that they will eventually overcome the obstacle(s) at hand [18].

A Canadian critical ethnographic study led by the lead author of this paper with nine young people who had recently exited homelessness revealed that supports for young people transitioning out of homelessness tended to focus on downstream tangible resources such as housing and welfare payments, with inadequate attention to upstream intangible resources such as identity capital [4, 16]. Findings generated from 10 months of intense engagement and 119 one-on-one interviews highlighted that, when faced with the reality of massive socioeconomic inequities and armed primarily with downstream supports, the young people grew exhausted and discouraged, in part because they had insufficient upstream resources to continue pushing toward their goal of socioeconomic inclusion. The study authors emphasize that the bolstering of identity capital is not meant to be a substitution for the reformation of socioeconomic inequities; rather, they advocate for helping young people “maximize their life chances and compete effectively given the realities in which they find themselves” [16 p124].

The overall aim of this study was to assess the feasibility of improving socioeconomic inclusion outcomes by bolstering identity capital among young people who had experienced homelessness. To be clear, we want to state at the outset that we are not proposing young people should “bootstrap” themselves out of homelessness; rather, our desire is to address the inequitable distribution of intangible, identity-based resources that are provided to most young people throughout their lives–a topic that has received almost no attention in the youth homelessness intervention literature.

## Methods

Nineteen young people (aged 18–26) who had transitioned out of homelessness within the past three years participated in a six-week, six-session program focused on building identity capital (sense of purpose and control, self-efficacy and self-esteem) and providing career direction. The program was designed and carried out by an established leadership and executive coaching centre in Toronto, Canada. The overarching theme of this program (“The Identity Project”) was on building skills related to emotional intelligence such as self-awareness and internal motivation, which were fostered through discussions on topics ranging from time management and organizational skills to goal setting and strategic career choices.

Two full-day workshops and four half-day group coaching sessions were held once a week for six weeks (six week, six session intervention). Importantly, the organization did not modify their program content for “homeless youth”; young people received the same messaging provided to global business leaders–many from Fortune 500 companies–in over 15 countries across North and South America, Europe and Asia [19]. Another intentional feature was that the weekly program was provided at the organization’s trendy, loft-like headquarters–an area not associated with homelessness (see [16] for more on identity and the underutilization of supports embedded in the homelessness sector). All of the young people were recruited by our community partner, Covenant House Toronto–Canada’s largest agency serving youth who are experiencing or have experienced homelessness [20] and provided with scholarships from a provincial poverty reduction initiative (Ontario Trillium Foundation Local Poverty Reduction Fund) to cover program and attendance costs. This study received ethics approval from the St. Michael’s Hospital Research Ethics Board (18–002) and all participants provided written consent. The study was prospectively registered on Clinical Trials.gov (ClinicalTrials.gov NCT03772522) after the intervention had concluded but while data collection was still in progress. The prospective registration was an unfortunate administrative oversight. There are no ongoing or related trials for this intervention at the time of this publication.

The study employed a mixed method prospective cohort hybrid design with a six-week, six-session intervention group (Group One; *n* = 8) and a delayed intervention group (Group Two; *n* = 11). Initially, we had hoped to conduct a pilot randomized controlled trial (RCT) with 30 participants. Given the pilot nature of the study, the sample size of 30 was based on feedback from the organization delivering the intervention regarding feasible group numbers, rather than aiming for statistical power. Unfortunately, by the time we received ethics approval we had less than three weeks to recruit participants as the organization’s busy schedule dictated a commitment to a start date several months in advance of the intervention. Given the small number of interested participants one week before the program start date, and after conferring with an experienced statistician, we decided to abandon the RCT design and begin the intervention with all interested participants (Group One) and continue recruiting for a delayed intervention group (Group Two).

**Fig 1:**
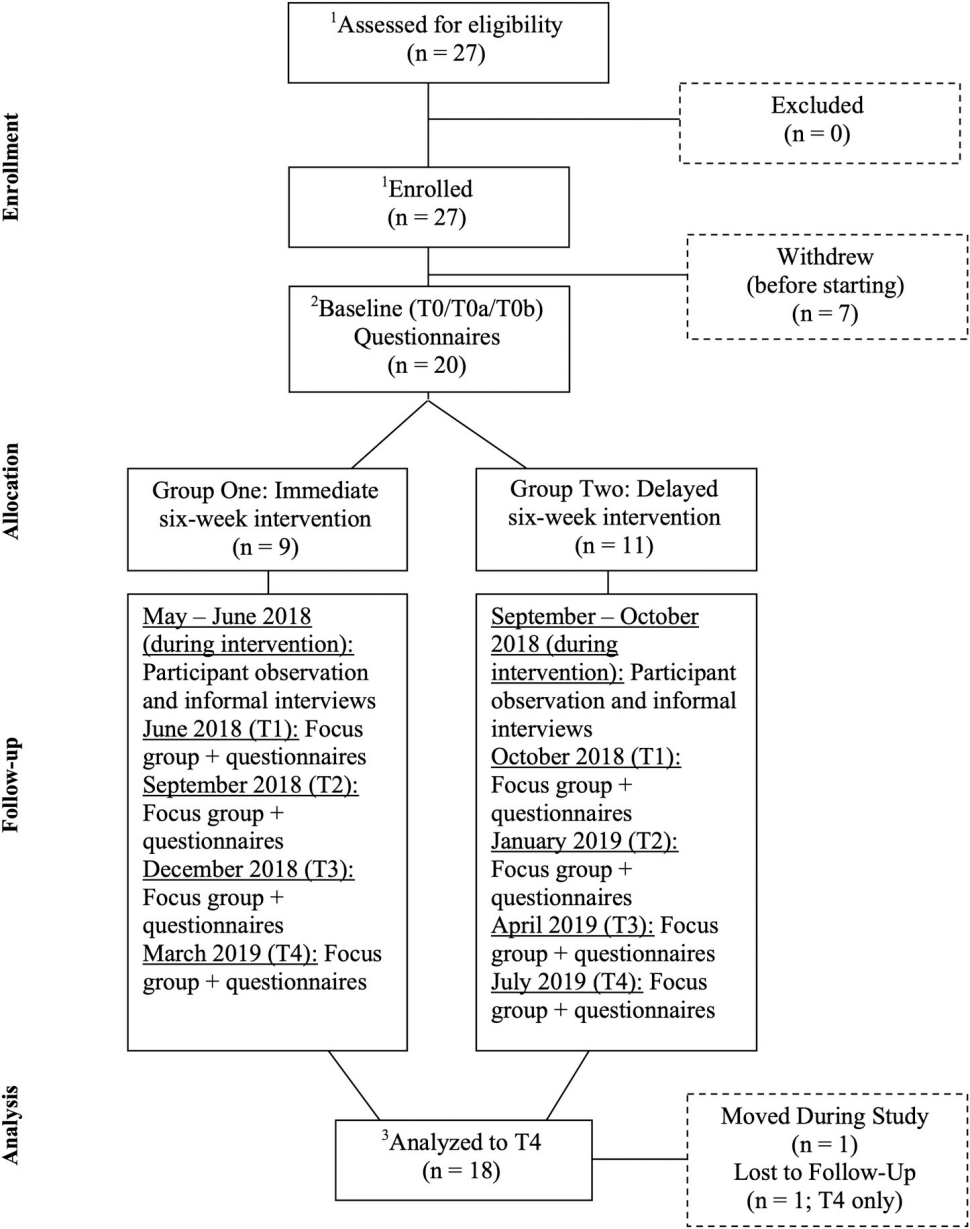
Flow of participation and analysis: The Identity Project. ^1^ Assessment of eligibility and enrollment continued while Group One began the intervention (no randomized assignment). ^2^ To compare six weeks of intervention (Group One) with six weeks of no intervention (Group Two), we conducted two baseline interviews with Group Two: one at initial study enrollment (T0a; mid-June–mid-July 2018 [rolling recruitment/enrolment]), and one six weeks later (T0b; late July–late August 2018 [second interview date dependent on date of initial enrolment/interview to ensure a six-week span between T0a and T0b]). ^3^ After Group Two received the same six-week, six-session intervention, Group One and Group Two participants (n = 19) were pooled and a single-arm study design was chosen to examine pre-post intervention changes during follow-up.

Participants in Group Two were offered the same six-week, six-session intervention as Group One, approximately three months after Group One had completed the intervention. While waiting to receive the intervention, Group Two received standard shelter-based, transition-related supports such as assistance finding housing and connection to employment and/or education. To compare six weeks of intervention (Group One) with six weeks of no intervention (Group Two), we conducted two baseline interviews with Group Two–one at initial study enrollment (T0a), and one six weeks later (T0b). After Group Two received the same six-week, six-session intervention, Group One and Group Two participants (*n* = 19) were pooled and a single-arm study design was chosen to examine pre-post intervention changes during follow-up. Participants in both groups were followed every three months for nine months post-intervention (Fig 1).

### Conceptual framework

**Fig 2:**
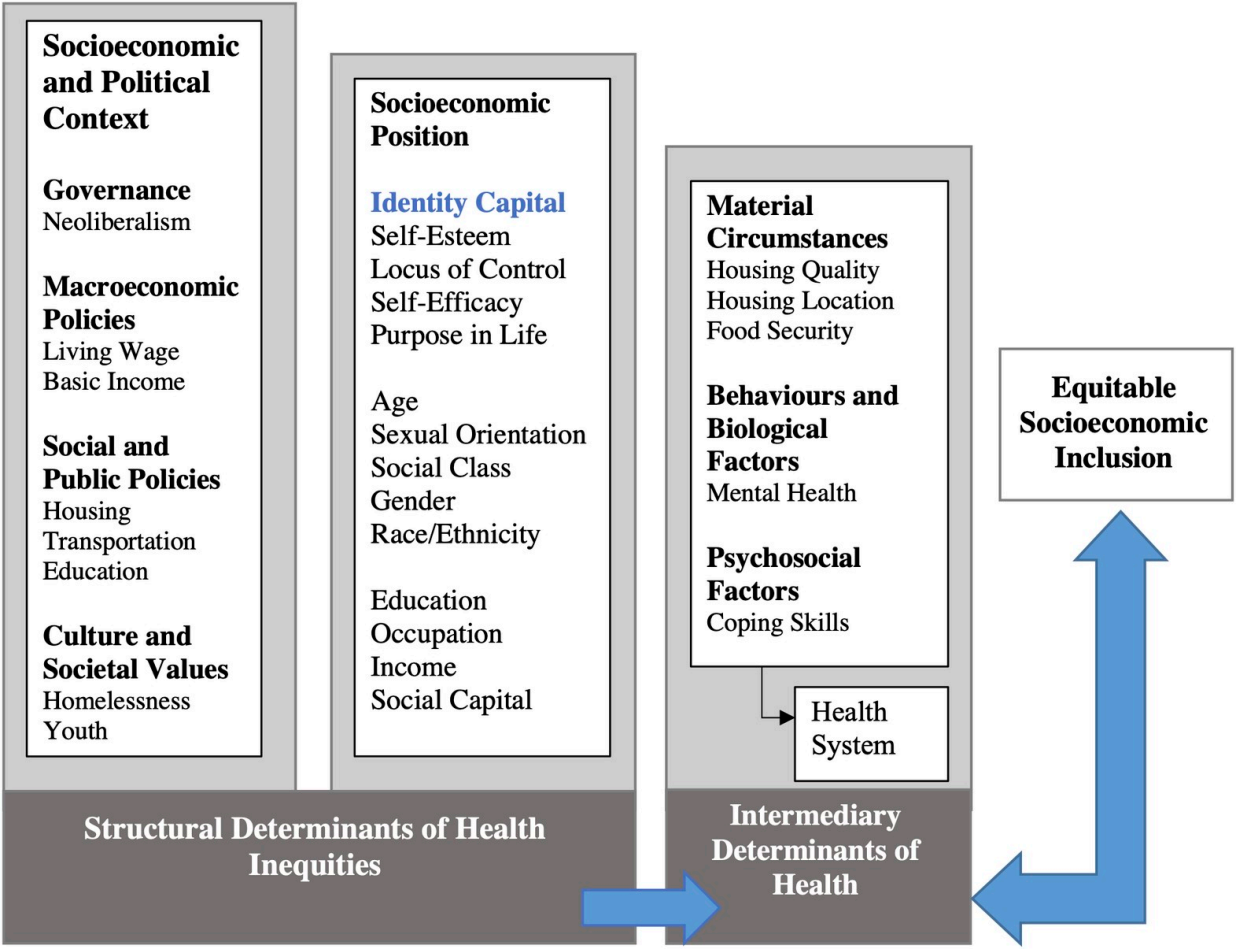
Equitable socioeconomic inclusion framework for youth experiencing homelessness. (Framework modified from the World Health Organization conceptual framework for action on the social determinants of health [23]).

Conceptually, the study was informed by an equitable socioeconomic inclusion framework for youth experiencing homelessness (Fig 2) developed during the aforementioned critical ethnographic study [16]. Specifically, we drew on the notion that, for youth who have experienced homelessness, identity capital is inherently and intrinsically linked to socioeconomic position and, ultimately, to equitable socioeconomic inclusion (see [16] for a more fulsome discussion on framework evolution, including critical social theoretical underpinning).

### Quantitative measures

Quantitative data (Table 1) for Group One were collected at five points in time over the course of 11 months: baseline (T0), immediately post-intervention (T1; six weeks), and at three (T2), six (T3), and nine months (T4) post-intervention. Quantitative data for Group Two were collected at six points in time over the course of 14 months: baseline(T0a), six weeks (T0b; to directly compare six weeks with no intervention to six weeks with an intervention), immediately post-intervention (T1), and at three (T2), six (T3), and nine months (T4) post-intervention. Because there is no clearly defined measure of socioeconomic inclusion [13], we drew from the work of our colleagues [11, 21, 22] and from our own work [4, 16] to identity proxy indicators of socioeconomic inclusion.

**Table 1:** Quantitative measures.

Instrument	Details
Baseline Demographic Questionnaire	We developed this 10-item questionnaire for this study.
Beck Hopelessness Scale [24]	This 20-item scale measures motivation, expectations, and feelings about the future (internal consistency α > .93).
Community Integration Scale [25]	This 11-item scale measures behavioral (e.g., participation in activities) and psychological (e.g., sense of belonging) aspects of community integration. This scale was used extensively in the multi-city Canadian At Home/Chez Soi study but psychometric properties have yet to be reported.
Education, Employment, Training, Employment, Income, and Housing Questionnaire	We developed this 7-item questionnaire for this study.
Rosenberg Self-Esteem Scale [26]	This 10-item scale measures global self-worth (internal consistency α .77 –.88).
Social Connectedness Scale-Revised [27]	This 20-item scale measures belongingness–the degree to which people feel connected to others (internal consistency α > .92).

#### Quantitative hypothesis and main outcomes

We hypothesized that, for the main quantitative outcomes of hopelessness, community integration, self-esteem, and social connectedness:

1. We would observe significant improvements in the mean scores of Group One (intervention group) compared to Group Two (delayed intervention comparison group) immediately post-intervention (T1 Group One compared to T0b Group Two).
2. We would observe significant improvements in the pooled mean scores of Group One and Group Two immediately post-intervention (T1) compared to baseline (T0/T0b).
3. Significant improvements in the pooled mean scores of Group One and Group Two would be sustained for at least three months (T2) post-intervention compared to baseline (T0/T0b).

### Qualitative measures

Qualitative measures consisted of participant observation, informal interviews, and focus groups. Over the six-week intervention period, study investigators NST and AW attended the weekly sessions as observers. During these sessions, NST took field notes, documenting perceptions about the way the workshops and group coaching sessions were being run, and how they are being taken up by the young people. NST also conducted informal interviews with the youth during this six-week participant observation period. These informal interviews acted as a form of data triangulation, allowing her to confirm inferences made through participant observation [28, 29]. The informal interviews were brief and conversational in nature and conducted at opportune moments during the workshops and group coaching sessions (e.g., during coffee or lunch breaks). Data generated during the informal interviews were documented contemporaneously and incorporated into the field notes.

#### Focus group data

We held eight separate focus groups for Group One and Group Two (four focus groups each), and all sessions were led by NST. Initially, it was our intention to conduct two semi-structured interviews with select key informants from Group One and Group Two, and two focus groups with Group One and Group Two (four total) over the follow-up period. However, given we had a smaller number of study participants than originally anticipated, and our observation that many participants grew close over the six-week intervention, we decided to interview the young people together and hosted four focus groups with Group One and Group Two (eight total) over the follow-up period. The focus group sessions were conducted immediately after the intervention and then every three months for nine months post-intervention. Everyone who participated in the intervention attended at least one focus group (the majority attended at least two), except for one young person who moved to another province immediately post-intervention. We had an average attendance of five young people at each focus group session. All focus groups were audio recorded and transcribed by AW who attended all the sessions.

### Quantitative analysis

To address the first question about whether Group One demonstrated a statistically significant difference in outcomes compared to Group Two during the time period where Group One was completing the program and Group Two had not yet started the program, we compared the difference in Group One main outcome variable values six weeks after the baseline interviews to the baseline values to the respective differences in Group Two values. To conduct the analysis, we used independent *t*-tests to compare the difference of differences and Cohen’s d measure of between-group effect size. Capturing effect size is especially relevant for studies with smaller sample sizes because a change in effect size can suggest an effect of the intervention even when results do not appear *statistically* significant [30]. To address the second and third questions about the extent to which the main outcome variables changed immediately after the intervention and over time, we conducted paired *t*-tests and Cohen’s d measure of within-group effect size on the outcome variables to report on changes between: 1) baseline (T0/T0b) and immediately post-intervention (T1); 2) baseline (T0/T0b) and three months post-intervention (T2); 3) baseline (T0/T0b) and six months post-intervention (T3); and 4) baseline (T0/T0b) and nine months post-intervention (T4). Statistical tests were two-sided with α < 0.05 considered significant. Statistical analyses were performed with IBM SPSS Statistics for Macintosh, Version 26.0 (Armonk, NY: IBM Corp).

### Qualitative analysis

Our analysis was guided by the equitable socioeconomic inclusion framework for youth experiencing homelessness (Fig 1). The framework operated like our conceptual “glasses”, influencing what we saw in the data. To further facilitate analytic depth and praxis-oriented, substantive interpretations, NST also drew on knowledge gleaned from her dual roles [31, 32] as a nurse practitioner and researcher who works primarily with young people who are experiencing or have experienced homelessness.

Prior to each focus group AW conducted a preliminary data analysis, reading the focus group transcripts (which she had also transcribed) multiple times, separating the data into coded segments, making analytic memos beside large portions of the transcripts, and identifying emerging themes [33, 34]. This preliminary analysis was discussed with NST and assisted with compiling new questions prior to each focus group session. Participants were also asked for their perspectives on the emerging interpretations at each focus group and these perspectives played key role in helping shape the analysis and contributed to the trustworthiness of the data [33, 35]. The web-based application Dedoose [36] was utilized to assist with sorting and coding the qualitative data.

## Results

### Demographic characteristics

The average age of participants was 23 years (Table 2). There were more self-identified females (58%) than males (42%). The majority (69%) identified as non-white. The majority of youth had completed high school (79%) and were receiving social welfare payments (79%). Almost half (48%) were employed. All of the youth were housed at baseline (an inclusion criteria) and, on average, participants had attempted to leave homelessness twice prior to participating in this study (note: for the purposes of this study, we considered living in transitional housing as exiting homelessness).

**Table 2:** Baseline demographic characteristics of participants in The Identity Project (N = 19).

Variable	Group Total (*n* = 19)	Group One (Immediate Intervention) (*n* = 8)	Group Two (Delayed Intervention) (*n* = 11)
	*n* (%)	*n* (%)	*n* (%)
Age (y), mean (SD)	22.9 (2.2)	22.5 (3.1)	23.2 (1.3)
Self-identified gender^1^			
Female	11 (57.9)	6 (75.0)	5 (45.5)
Male	8 (42.1)	2 (25.0)	6 (54.5)
Ethnoracial group			
Black	7 (36.8)	4 (50.0)	3 (27.3)
White	4 (21.1)	2 (25.0)	2 (18.2)
Asian	4 (21.1)	1 (12.5)	3 (27.3)
Different choice	4 (21.1)	1 (12.5)	3 (27.3)
Highest level of education			
Less than high school	4 (21.1)	2 (25.0)	2 (18.2)
Completed high school	8 (42.1)	2 (25.0)	6 (54.5)
Some or completed post-secondary	7 (36.8)	4 (50.0)	3 (27.3)
Social assistance			
Non-disability benefit (Ontario Works)	8 (42.1)	4 (50.0)	4 (36.4)
Disability benefit (Ontario Disability Support Program)	7 (36.8)	2 (25.0)	5 (45.5)
None	4 (21.1)	2 (25.0)	2 (18.2)
Employment			
Full-time (≥ 30 hours/week)	3 (15.8)	1 (12.5)	2 (18.2)
Part-time (< 30 hours/week)	6 (31.6)	4 (50.0)	2 (18.2)
Current living situation			
Market housing	7 (36.8)	2 (25.0)	5 (45.5)
Transitional housing^2^	3 (15.8)	3 (37.5)	1 (9.1)
Subsidized/social housing	8 (42.1)	2 (25.0)	5 (45.5)
With parents^3^	1 (5.3)	1 (12.5)	0 (0.0)
Number of attempts at leaving homelessness			
<2	9 (47.4)	2 (25.0)	7 (63.6)
> = 2	10 (52.6)	6 (75.0)	4 (36.4)
Median (range)	2 (0^3^−5)	2 (0^3^−3)	1 (1–5)

^1^Participants were given the option of choosing non-binary; none chose this.

^2^Affiliated with homelessness sector.

^3^One participant had a history of homelessness service use and housing precarity but had never experienced homelessness.

None of the youth who began the intervention dropped out of the program, with the exception of one participant who moved across the country and was unable to continue. All of the youth attended at least three intervention sessions. None of the youth were lost to study follow-up, with the exception of one youth who did not participate in the nine-month post-intervention data collection due to personal challenges.

### Quantitative results

Differences in Group One and Group Two baseline scores were not statistically significant with the exception of the physical component of the Community Integration Scale, for which Group Two scored higher at baseline (S1 Table). Immediately after participating in the intervention, Group One (*n* = 8) had better score improvements in hope, physical and psychological community integration, social connectedness, and self-esteem compared to Group Two (*n* = 11), which had not yet begun the program (Table 3). Of note, Group One had statistically significant improvements and large to very large effect sizes in self-esteem (*d* = 1.2) and physical community integration (*d* = 1.8) compared to Group Two. There were no statistically significant improvements or changes to effect sizes for psychological community integration and social connectedness.

**Table 3:** Means (M), standard deviation (SD), difference in means (DM), and between-group effect sizes (Cohen’s *d*) of Group One immediately after receiving six weeks of the intervention compared to Group Two after six weeks of no intervention.

Outcome Variable	Group One (Immediate Intervention) (*n* = 8)			Group Two (Delayed Intervention) (*n* = 10)^1^			
	T0	T1	DM	T0a	T0b	DM	*d*
	M (SD)	M (SD)	(SD)	M (SD)	M (SD)	(SD)	
BHS	4.27 (3.28)	2.63 (3.46)	-1.64 (2.52)	6.70 (4.08)	6.20 (3.82)	-0.50 (3.27)	0.38
CIS-Phy	2.50 (1.20)	3.89 (1.11)	1.39 (0.97)	4.42 (2.12)	3.30 (2.31)	-1.12 (1.66)	1.79*
CIS-Psy	11.63 (4.27)	11.88 (4.36)	0.25 (4.23)	12.90 (3.98)	13.00 (3.80)	0.10 (2.13)	0.05
SCS-R	74.00 (13.37)	75.75 (25.41)	1.75 (18.89)	70.00 (10.10)	73.20 (14.53)	3.20 (9.33)	0.10
RSES	16.66 (2.92)	22.00 (4.87)	5.34 (5.10)	17.65 (4.90)	18.50 (6.42)	0.85 (2.52)	1.16*

T0: baseline; T1: immediately post-intervention; T0a: baseline a (at enrolment); T0b: baseline b (six weeks later; see Fig 1).

DM: difference in T0 and T1 means (Group One), and difference in T0a and T0b means (Group Two).

d: effect size between the differences in means of Group One and Group Two; 0.2 = small, 0.5 = medium, 0.8 = large.

*Independent-samples t-test of the differences in means of Group One and Group Two, p < 0.05.

^1^One participant in Group Two was enrolled after the six-week period of no intervention (but before Group Two began) and was excluded from this analysis.

In the pooled Group One and Group Two scores immediately post-intervention (Table 4), improvements to hope, community integration, social connectedness, and self-esteem remained, with statistically significant improvements and moderate effect sizes in physical community integration (*d* = 0.60) and self-esteem (*d* = 0.62) compared to baseline. Additionally, there were small effects seen in hopelessness (*d* = -0.46) and psychological community integration (*d* = 0.20) compared to baseline; however, these were not statistically significant. Also, there was no statistically significant improvement or changes in effect size for social connectedness.

**Table 4:** Means (M), standard deviation (SD), and within-group effect sizes (Cohen’s *d*) for participant outcomes.

Outcome Variable	T0/T0b	T1		T2		T3		T4	
	(*n* = 19)	(*n* = 19)		(*n* = 19)		(*n* = 19)		(*n* = 18)^1^	
	M (SD)	M (SD)	*d*1	M (SD)	*d*2	M (SD)	*d*3	M (SD)	*d*4
BHS	5.59 (3.68)	4.12 (3.92)	-0.46	4.36 (3.59)	-0.40	3.64 (3.37)	-0.73*	3.72 (3.75)	-0.60*
CIS-Phy	2.84 (1.89)	3.64 (1.57)	0.60*	3.73 (2.14)	0.52*	3.68 (1.63)	0.51*	3.39 (1.85)	0.32
CIS-Psy	12.16 (3.98)	12.79 (4.25)	0.20	12.53 (2.80)	0.09	12.21 (4.04)	0.01	11.94 (3.62)	-0.07
SCS-R	72.26 (14.39)	75.42 (19.23)	0.19	78.47 (14.92)	0.42	77.32 (16.21)	0.33	77.49 (15.19)	0.37
RSES	17.70 (4.97)	20.37 (6.40)	0.62*	19.32 (5.71)	0.29	21.53 (6.70)	0.71*	20.11 (4.63)	0.53*

T0/T0b: pre-intervention; T1: immediately post-intervention; T2: three-months post-intervention; T3: six-months post-intervention; T4: nine-months post-intervention.

d: within-group effect size as compared to baseline; 0.2 = small, 0.5 = medium, 0.8 = large.

*Paired-samples t-test, p < 0.05.

^1^We were unable to obtain data for one participant at the T4 follow-up period.

We observed improvements over time in the pooled Group One and Group Two scores–particularly in the areas of hope and self-esteem (Table 4). Hopelessness levels continued to decrease post-intervention and at six- and nine-months, a statistically significant, medium effect size increase was observed (*d* = -0.73 and *d* = -0.60 respectively). The initial increase in physical community integration observed immediately post-intervention compared to baseline was sustained until six months post-intervention (*d* = 0.51), after which a small decrease was observed at nine months (*d* = 0.32), making it no longer statistically significant. Psychological community integration remained relatively stable during the follow-up period with no changes to effect size beyond immediately post-intervention and never reaching statistical significance. Although changes in social connectedness did not reach statistical significance, a slight upward trend towards improvement was observed; a small effect was seen at the three month follow-up period (*d* = 0.42) and this effect was maintained for the remainder of the study. A statistically significant, moderate effect was seen in self-esteem at six months (*d* = 0.71) and nine months (*d* = 0.53), showing that the initial increase in self-esteem immediately post-intervention was maintained for the duration of the nine month follow-up.

At nine months post-intervention, three youth had completed high school and three youth–one from the group of high school completers–began post-secondary education (Table 5). There were no overall improvements in the total number of participants in employment or training enrollment compared to baseline. The nine-month post-intervention average after-tax employment income of $1,146 CAD (SD = $643) was still below Canada’s low-income after-tax measure (“poverty line”) of $2,015 CAD/month for a single adult [37], and the majority (72%) of young people continued to receive social assistance payments. None of the young people returned to homelessness during the study.

**Table 5:** Education, employment, training, employment income, and housing outcomes.

Variable	T0/T0b	T1	T2	T3	T4
	(*n* = 19)	(*n* = 19)	*(n* = 19)	(*n* = 19)	(*n* = 18)^1^
	*n* (%)	*n* (%)	*n* (%)	*n* (%)	*n* (%)
Enrolled in secondary education	4 (21)	4 (21)	3 (16)	3 (16)	1 (6)^2^
Enrolled in post-secondary education	4 (21)	5 (26)	6 (32)	6 (32)	7 (39)
Employed full-time (≥30 hours/week)	3 (16)	2 (11)	3 (16)	2 (11)	2 (11)
Employed part-time (< 30 hours/week)	6 (32)	4 (21)	4 (21)	7 (37)	5 (28)
Training	1 (5)	3 (16)	3 (16)	2 (11)	1 (6)
Average (CAD) monthly employment income (SD)	$1356 ($864)	$1083 ($881)	$1843 ($950)	$1227 ($712)	$1146 ($643)

T0/T0b: pre-intervention; T1: immediately post-intervention; T2: three-months post-intervention; T3: six-months post-intervention; T4: nine-months post-intervention.

^1^We were unable to obtain data for one participant at the T4 follow-up period.

^2^Three youth completed secondary education between T1 and T4.

### Qualitative results

The improvements we witnessed in the quantitative data–particularly the longer term improvements to self-esteem and hopelessness–were also evident in our qualitative findings. Two major themes came from the analysis of our focus group discussions: vision for life and reconstructing identity.

#### Vision for life

Many participants shared how experiences of homelessness can undermine self-esteem, reinforce identities of exclusion, and distort beliefs about what one is capable of becoming. For example, even though we thought we had made the program design clear (i.e., concepts taught to business leaders), several participants shared that, because they were recruited by a homelessness-serving agency, they had low program expectations, anticipating a “homelessness” or “mental health” program. In other words, they had internalized that young people experiencing homelessness are provided with *certain kinds* of programming.

*“I don’t feel like the shelter is what I represent*. *I felt like I was downgraded*. *Going to this program helped boost up my self-esteem again*.*”* (Dominic, Group 1, Focus Group 2)

An important precursor to a renewed vision for life was the creation of a vision board. On the first day of the program, participants created a vision board made from magazine clippings that depicted their “ideal” life. During the follow-up period, many young people noted they were keeping their vision boards nearby as hopeful inspiration. Notably, at our final focus groups, several participants shared that their vision board had moved from a physical location (e.g., beside their beds) to something they could “see” mentally as they went about their day, serving as a reminder of essential program takeaways.

*“When I look at [the vision board]*, *it reminds me of all the things I did when I was in [the program]…it isn’t just a vision board I am seeing*, *it is everything I learned with it that I see*.*”* (Shahana, Group 1, Focus Group 4)

The program also helped youth (re)gain a sense of control. Throughout the six-week program, participants were asked to imagine where they were located in their “car of life.” They were encouraged to move from the passenger seat (or back seat) into the driver’s seat and to consider who needed to be removed from (or added to) their car of life. They were also challenged to envision where they were headed.

*“My main takeaway is that I am the master of my own future*. *I feel lots more in control*. *I’m in the driver’s seat*.*”* (Nayah, Group 2, Focus Group 1)

#### Reconstructing identity

Participants were told that business leaders are looking for young people who are tenacious and gritty–qualities often acquired by those who have persevered through adversity–and too often in short supply in today’s workforce. This reconceptualizing of the experience of homelessness–from deficit to asset–resonated with many program participants and served to reconstruct how they saw themselves and their potential to enact change.

*“I think [the program] will help us in our future…step-by-step*. *It will give us confidence to do things like speak in front of people…I loved learning new skills…I am still nervous [to speak in front of others] but it’s better…I think the future is going to be okay*.*”* (Bella, Group 2, Focus Group 1)

Many shared how they were gaining the confidence to take care of themselves as opposed to relying on others (often social service providers) for support. This internal (vs. external) sense of control seemed to give participants confidence to move forward in their transition away from homelessness.

*“Before*, *I needed to have support from my worker or some other person [to make decisions]*. *But now*, *I’m doing it by myself… Now I see myself in the car*, *like in the driver’s seat*, *and I feel proud of myself*. *Like*, *I see [added emphasis] myself there*.*”* (Katherine, Group 2, Focus Group 2)

Akin to the vision board being a precursor to a renewed vision for life, the creation of a daily scheduled was a precursor to reconstructing identity. Participants created a detailed daily schedule (routine) during the program, and many spoke about the importance of this even nine-months post-intervention. Importantly, they explained that creating and sticking to a daily schedule is much easier *after* you have a vision for your life.

*“It [new skills] felt more tangible–there was more agency*, *especially with the daily routine*. *I remember when I was in crisis*, *[previous counsellors] said*, *‘Go to [support] group*, *go exercise*, *go do this*, *go do that*.*’ But it wasn’t like I was motivated to do these things*. *It was just*, *‘this is how you’ll get better*,*’ versus here [at the program] it’s*, *‘This is how you’ll live a life that’s good for you*.*’”* (April, Group 1, Focus Group 3)

Many youth shared that they appreciated the normalizing (vs. pathologizing) of strategies they needed to learn and spoke of the importance of framing new skills as something one needs “to have a better life” vs. “to get better.” In other words, the skills they were learning were skills *all* people–even “successful” business folks–need, not “treatment” required by someone who was “sick.” Notably, most youth acknowledged they were more likely to accept key program messaging because it was imparted by an organization operating outside the homelessness sector. For example, many of the youth followed the organization on social media and took delight when the CEO–the person leading all their sessions–posted pictures of herself delivering the same program concepts to national and international business leaders.

## Discussion

As we stated at the outset of this paper, interventions designed with the primary aim of addressing inclusion-related challenges for youth who are experiencing or have experienced homelessness are rare. Keeping the exploratory nature of this study and our small sample size in mind, we are cautiously optimistic that targeting identity capital might be a promising approach–alongside other tangible determinants of health such as housing and employment–to helping youth transition out of homelessness and achieve meaningful socioeconomic inclusion.

Immediately after participating in the six-week, six-session intervention, Group One had statistically significant improvements and large to very large effect sizes in self-esteem and physical community integration compared to Group Two, which had not yet begun the intervention. This was not a surprise given many interventions with this population demonstrate short-term positive impacts [10]. However, in the pooled analysis, small to moderate effect sizes in hopelessness, physical community integration, and self-esteem were observed at all post-intervention time points, with statistically significant improvements and moderate effect sizes in hopelessness and self-esteem at six- and nine-months post-intervention. These latter time points are important as reviews of interventions with young people who are experiencing or have experienced homelessness show longer-term, post-intervention follow-up is rarely achieved [10, 38]. Also, the sustained improvements specific to hopelessness and self-esteem are noteworthy given previous longitudinal studies with youth exiting homelessness have shown that these outcomes tend to get worse over time [3, 4]. Finally, the improvement to hope is especially encouraging given this domain is a key feature of recovery-oriented practice [15], which underpins the Housing First philosophy [39].

It is challenging to situate our study among other interventions studies with this population because of the heterogeneity of the studies (e.g., different outcomes and self-report scales) and because the majority these studies include–often exclusively–young people who are still experiencing homelessness (see recent review by Morton et al. [10]). Additionally, as noted by our study participants, interventions with this population tend to primarily focus on treating mental health or targeting behavior change (e.g., substance use) [7, 9, 10], not specifically targeting identity capital. That said, there are some transferrable comparisons. For example, a feasibility study of a six-month comprehensive intervention consisting of transition-focused case management, individual/group counseling, and peer support with 31 formerly homeless young people also included measures of hope and community integration (baseline and immediately post-intervention); however, there were no statistically significant or effect size improvements in these outcomes compared to baseline [21]. It is important to note that, unlike our study, participants did make gains in the area of employment, with 15 youth obtaining new or improved positions. The qualitative data from this mixed methods intervention highlighted the importance of transition-focused case management–something we did not offer in our intervention–in assisting with connections to employment [21].

Authors of the aforementioned six-month feasibility study note they incorporated promising mental health interventions with young experiencing homelessness such as Dialectical Behavioral Therapy (DBT) into their comprehensive intervention. McCay et al. [22] was able to demonstrate statistically significant improvements in several mental health outcomes, including hopelessness and self-esteem, at 10 weeks post-intervention for 60 young people (the majority were living in homeless shelters/transitional housing) who participated in a 12-week DBT intervention. A recent review of randomized controlled trials and systematic reviews on mental health interventions for youth experiencing homelessness found that Cognitive Behavioral Therapy (CBT) was a promising intervention for youth experiencing depression, with the authors noting that DBT principles were incorporated into some of the CBT interventions [40]. What is interesting about these encouraging DBT/CBT approaches relative to our study is that the person leading our intervention was a psychotherapist and acknowledged incorporating DBT/CBT principles (e.g., developing skills to manage emotion and respond to challenging situations more effectively) into the program content; however, she did so in a non-pathologizing/“non-treatment” manner–viewing herself more “coach” than therapist–a strategy very much appreciated by our program participants.

The qualitative data generated from focus groups with study participants was an important addition to this study and aligns with increasing emphasis to draw on the wisdom of those who have experienced homelessness to help researchers operationalize outcomes beyond housing stability [6, 13, 14]. It was interesting to explore with participants how the program’s particular emphasis on life purpose and being in control–key components of identity capital–manifested in measurable and sustained improvements in hopelessness and self-esteem. In particular, the antecedents to having a sense of purpose (vision board) and being in control (daily schedule) very much align with emerging literature from the field of occupational science on the importance of addressing the sense of boredom and meaninglessness that often plagues those who are experiencing or have experienced homelessness [41]. In addition, intervening on personal control has shown promise as a homelessness prevention strategy. A six-month strengths-based intervention targeting personal control with youth experiencing homelessness and struggling with substance use demonstrated that personal control mediated the effect of cumulative risks (e.g., depression, childhood sexual and physical abuse, and injection drug use) on housing stability over the nine-month follow-up period [42].

It was encouraging to observe three young people finish secondary education, three enroll in post-secondary education (two were different from the three that finished secondary education), no one dropping out of secondary or post-secondary education, and no one returning to homelessness during the nine-month follow-up period. A recent pan-Canadian survey of 1,100 youth who were experiencing or had experienced homeless found that only a small number had completed secondary education (21%) or participated in post-secondary education (12%), a large number (53%) had dropped out of secondary education, and the majority (76%) reported at least two separate episodes of homelessness (and of those, 37% reported more than five episodes of homelessness) [43]. While we cannot say that our education- and housing-related findings were due to the intervention, these trajectories were encouraging and an important contribution given the significant gap in the peer-reviewed youth homelessness intervention literature on targeting these outcomes [10]. In addition, analysis of the aforementioned pan-Canadian survey showed that participants who reported more episodes of homelessness were significantly more likely to self-identify as a “homeless youth” [44], giving credence to our hypothesis on the relationship between identity capital and socioeconomic inclusion, and highlighting the underexplored link between identity and chronic homelessness.

While we did observe encouraging changes in some of the internal (identity capital) and external (sustained/improved education and stable housing) resources we posit are required for equitable socioeconomic inclusion, the intervention was not able to move the young people out of poverty. This aligns with our observations of intermittent fluctuations but ultimately no positive change in employment and training between baseline and nine months post-intervention (Table 5). For one participant, moving from employed to unemployed represented a conscious decision to better focus on post-secondary education; however, for the rest of participants, there was a notable lack of meaningful employment and training opportunities. Moreover, given the majority of youth were receiving social assistance payments, employment income “claw backs” (e.g., those on Ontario Works were deducted 50 cents for every dollar earned over $200 of monthly employment income) [45] and worry over losing assistance-related benefits (e.g., free eye exams) may have been a disincentive to seeking employment.

Arguably, the three young people who finished high school and two who enrolled in post-secondary education (three youth enrolled in post-secondary education but only two were different from the high school completers) during the course of this study may be able to leverage their education to help move them out of poverty, but this outcome will take longer to be realized. When these secondary quantitative outcomes related to education, housing, employment income, employment, and training are placed within the Equitable Socioeconomic Inclusion Framework (Fig 1), we see the inextricable link between identity capital and other external, tangible factors that move youth toward socioeconomic inclusion such as occupation (i.e., living wage employment opportunities) and social capital (e.g., using relationships as “currency” to procure meaningful employment)–elements we did not provide with this intervention.

### Limitations

Our mixed methods approach, nine -month post-intervention follow-up, and 95% intervention and study follow-up participation rates are important strengths; however, this study does have notable limitations. First, this study was not adequately powered; causal inferences must be made with caution. Our small sample size also means the findings cannot be generalized; however, the synergistic effect of combining the quantitative findings with the qualitative data creates important conceptual insights that may be transferrable to similar contexts. Second, this study lacked a control group beyond immediately post-intervention. Thus, it is conceivable that the participants may have improved in our main study outcomes over time without the intervention; however, as noted previously, the limited longitudinal research with young people exiting homelessness does not bear this out. Third, while our participants were similar to youth still experiencing homelessness in many ways (e.g., majority on social assistance, had made at least two attempts to exit homelessness, and identified as belonging to a racialized group), they were more highly educated, with only 21% not completing high school at baseline compared to 53% in the aforementioned pan-Canadian survey that included youth still experiencing homelessness [43]. Plausibly, a group of young people still experiencing homelessness and with less years of formal education might not have had similar positive outcomes, and this demographic distinction is very important to keep in mind. For example, it may be that the ontological security associated with housing stability is required before young people are able to take up and benefit from the type of intervention we describe in this paper. Finally, while we made our exclusion criteria deliberately small, young people wrestling with challenges related to mental health and substance use may have not enrolled in the study as they might have been unable to commit to the six-week, six-session attendance. Therefore, this intervention may not be applicable to young people struggling with these challenges. That said, many young people informally shared their mental health challenges with us over the nine-month follow-up period, so we are confident that our study did include young people grappling with their mental health.

### Implications

The findings from this exploratory pilot study signal important implications in terms of how we conceptualize and assist young people transitioning out of homelessness. The inherent dignity associated with being coached (i.e., being cheered on; fostering purpose/control) versus being managed (i.e., case management; focus on pathologies) out of homelessness is a key takeaway from this study and warrants further investigation.

Those seeking to assist young people transitioning out of homelessness should consider paying more attention to the internal, intangible resources needed to navigate the mainstream such as identity capital, in addition to providing external, tangible resources such as housing and living wage job opportunities–both types of resources are needed to prevent homelessness from reoccurring. In other words, our aim–indeed our measure of success–should be to ensure young people achieve socioeconomic inclusion in the mainstream, not simply assisting them to survive and remain dependent on the social service sector.

The development of an identity capital checklist may be a beneficial prompt for those working in the homelessness sector to explore and act upon outcomes beyond housing stability. Moreover, collaborating outside the homelessness sector to act on outcomes related to identity capital could prove less stigmatizing and enhance a sense of societal inclusion. Additionally, given the emerging literature from the occupational science domain on the importance of targeting boredom and meaninglessness for people experiencing homelessness, the incorporation of an occupational therapist into transition-related supports may prove beneficial.

We believe our promising findings warrant a sufficiently powered larger trial and have secured funding to do so. We also plan to incorporate a virtual component to enhance access–a factor even more salient since the COVID-19 pandemic began. As previously noted, we were limited in our ability to foster–at least in the relative short-term–socioeconomic inclusion. On average, there were no improvements to poverty-level incomes, and psychological community integration and social connectedness never reached statistical significance at any point during the study. Thus, there is an urgent need to connect young people exiting homelessness with the financial and social capital needed for meaningful socioeconomic inclusion.

Some of the authors on this paper are involved in a pilot RCT where young people exiting homelessness are provided with rent subsidies and mentorship for two years [46]. All of the young people are provided rent subsidies (financial capital), while the intervention group is provided with mentorship. We are seeking to understand whether the bolstering of social and identity capital through mentorship will lead to better socioeconomic outcomes relative to the control group who are only receiving financial support. Our preliminary unpublished qualitative findings suggest that, although the intervention group is achieving some sense of ontological security through stable housing, mentorship may not be targeting identity capital as much as we had hoped; therefore, our intention for next iteration of the study is to incorporate a more explicit identity capital intervention such as the one describe in this paper alongside rent subsidies.

## Conclusion

When viewed within the Equitable Socioeconomic Inclusion Framework for Youth Experiencing Homelessness (Fig 1), our findings align with and expand upon the notion of what it means to provide recovery-oriented care to youth transitioning out of homelessness. Identity-informed care is inherently connected to recovery-oriented care–both highlight the fundamental value of gaining a sense of personal control and mastery to heal and move forward.

Over the course of almost one year (baseline to nine-months post-intervention), we witnessed young people grow in their self-esteem, sense of purpose, feelings of hope, and a belief that they had the ability to control their own destinies. Having their past consistently conceptualized as being an asset rather than a liability–especially by someone who works outside the homelessness sector–was a novel notion for the majority of participants and seemed to heal some of the identity-damaging messages they had explicitly (e.g., childhood trauma) and implicitly (e.g., living in a homeless shelter) received and internalized.

A crucial takeaway that bears repeating is that young people shared the importance of gaining a sense of purpose and control *before* being able to envision and move toward a better future. In other words, it is challenging to keep motivated if you inherently do not believe you have the capacity to succeed. We hope the findings from this exploratory study will make a meaningful contribution to the emerging discourse on how to help young people who have experienced homelessness move beyond housing stability and achieve equitable socioeconomic inclusion.

## Supporting information

S1 Table Means (M), standard deviation (SD), and independent-samples t-test for participant outcomes at baseline. (DOCX)

S1 Checklist TREND checklist. (PDF)

S1 File Study protocol. (PDF)
