# Supplementary material for: Tackling exclusion: A pilot mixed method quasi-experimental identity capital intervention for young people exiting homelessness
Source: PLoS One. 2021 Aug 20;16(8):e0256288. doi: 10.1371/journal.pone.0256288 (PMC8378743; doi:10.1371/journal.pone.0256288)
Supplement: S1 Table — (DOCX) [file pone.0256288.s001.docx]

**S1 Table. Means (M), standard deviation (SD), and independent-samples t-test for participant outcomes at baseline.**

| **Outcome Variable** | **Group One (Immediate Intervention)**  **(T0)**  **(*n* = 8)** | **Group Two (Delayed Intervention) (T0a)**  **(*n* = 10)** | ***p*-value** |
| --- | --- | --- | --- |
|  | **M (SD)** | **M (SD)** |  |
| BHS | 4.27 (3.28) | 6.70 (4.08) | 0.19 |
| CIS-Phy | 2.50 (1.20) | 4.42 (2.12) | 0.03 |
| CIS-Psy | 11.63 (4.27) | 12.90 (3.98) | 0.52 |
| SCS-R | 74.00 (13.37) | 70.00 (10.10) | 0.48 |
| RSES | 16.66 (2.92) | 17.65 (4.90) | 0.62 |
