# Supplementary material for: Tackling exclusion: A pilot mixed method quasi-experimental identity capital intervention for young people exiting homelessness
Source: PLoS One. 2021 Aug 20;16(8):e0256288. doi: 10.1371/journal.pone.0256288 (PMC8378743; doi:10.1371/journal.pone.0256288)
Supplement: S1 File — (PDF) [file pone.0256288.s003.pdf]

The Identity Project: An Evaluation of an Identity Capital Intervention for Young People Transitioning Out of Homelessness

1. Background and Rationale

Young people comprise almost 20% of the homeless population in Canada.[1] It is estimated between 35,000 and 40,000 Canadian youth (aged 13 – 25 years) are homeless at some point during the year and at least 6,000 on any given night.[2] In Toronto, an estimated 1,000 – 2,000 youth find themselves searching for a place to sleep on any given night.[3]

Transitioning off the Streets

While we know a great deal about the risk factors associated with young people entering and remaining entrenched in street life (e.g., intergenerational poverty, childhood abuse, inadequate education, and limited employment opportunities), we know much less about how to facilitate and sustain transitions off the streets for youth experiencing homelessness.[4-6] In fact, in the peer-reviewed literature, there have only been three published longitudinal studies examining the life-trajectories of formerly homeless young people who were all housed at baseline,[5,7-8] and no published rigorous interventions targeting housing outcomes, life-trajectories, quality of life, or social integration for young people experiencing homelessness.[9-11] Understanding how to create successful pathways out of homelessness is crucial, because once youth become entrenched in street life, it becomes much harder for them to exit homelessness and escape a life of poverty.[12-15]

The scarcity of evidence on this issue means there are critical gaps in our knowledge about how best to help homeless young people move forward in life after they become housed. For example, results from a recent longitudinal mixed method study of 51 formerly homeless youth living in Toronto and Halifax – led by Dr. Sean Kidd and colleagues and one of the most rigorous investigations to date – showed that obtaining stable housing did not necessarily translate into a sense of belonging or connection to mainstream society.[5] While study participants demonstrated incredible commitment, the structural barriers they experienced during the transition process (e.g., inadequate education and underemployment) led to fragile identities (i.e., self-concept) as they experienced a significant decline in hope

and a sense of being 'stuck'.[5,16,17] Youth described feeling unprepared for and overwhelmed by the realities associated with the transition process, undermining their sense of belonging and confidence in achieving larger life goals.[5,16,17]

Building on the Kidd et al. study, Dr. Naomi Thulien, a postdoctoral fellow at the Centre for Urban Health Solutions (and co-investigator in this evaluation), spent 10 months following nine young people who had left the shelter system and moved into market rent housing in Toronto.[8,18] The study – believed to be the first of its kind – captured the crucial role identity plays in the transition away from homelessness. Despite their remarkable determination, study participants lacked sufficient **identity capital**[19] – conceptualized as a sense of purpose and control, self-efficacy, and self-esteem – to help them press on in the face of significant structural inequities. Moreover, the well-intentioned supports offered by social service agencies were underutilized and limited in their effectiveness because the supports tended to be deficit-focused with minimal attention to fostering identity capital. Additionally, the provision of these supports within the social service sector was stigmatizing because it reminded the young people of their old identities as homeless youth. And while most of the study participants appeared successfully housed, they are described as living in chronic precarity, trapped inside a "hamster wheel of poverty" (p. 193) with little insight into how to move forward and at high risk of returning to homelessness.[18] Thulien asserts that failure to understand and incorporate identity capital into transition-related supports may result in a poor return on investment as these supports are likely to be ineffective, underutilized, or rejected outright.

Identity-Based Interventions

One notable identity-based intervention is a randomized controlled trial that targeted 264 eighth graders from high-poverty and high-unemployment neighborhoods in Detroit, Michigan.[20] Participants who attended the seven-week intervention aimed at aligning the students' current identities with achievable positive adult identities, demonstrated improved outcomes on measures pertaining to academics, mood, and behavior, which was sustained through the two-year follow-up. The authors suggest brief identity-based interventions can have significant effects on outcomes.[20]

Despite being suggested as a promising approach,[19,21] we know of no other interventions (nationally or internationally) that are specifically targeting the enhancement of identity capital for formerly homeless young people. We are aware that, in partnership with the Centre for Addiction and Mental Health, Dr. Kidd and colleagues have built on their previously mentioned research and developed the Housing Outreach Program-Collaboration (HOP-C) – a multi-faceted intervention that offers mental health and peer supports alongside transitional case management to youth transitioning off the streets.[22] What distinguishes the proposed intervention from the HOP-C intervention is that the proposed intervention uniquely and strategically targets identity, is significantly less costly, and is offered outside traditional and potentially stigmatizing social service settings. Given the limited evidence-based interventions for youth transitioning out of homelessness, we believe there is a critical need for evaluations that compare findings based on different approaches, reflecting the diversity of the population and their corresponding needs.

Overview of Proposed Intervention

The proposed intervention is based on two premises: 1) identity is socially constructed and malleable; and 2) people act in identity-congruent ways.[19,23-24] Thus, the intervention aims to change socially constructed life trajectories by providing identity-enhancing resources to formerly homeless young people. Thirty young people (aged 18 – 26 years) who have transitioned out of homelessness within the past three years will be offered scholarships to participate in a **six-week intervention** that focuses on building identity capital and providing career direction. The intervention will be **designed and carried out by dk Leadership[1]** – an established, highly successful leadership and counseling centre in Toronto with a track record of significantly improving the life trajectories of teens and adults.[25] Importantly, study participants will be incorporated into the centre's current programming, meaning the intervention will be held at a location not associated with homelessness. Study participants will be **collaboratively recruited by dk Leadership and Covenant House Toronto** – Canada's largest agency for street-involved and homeless youth[3] – and a concerted effort will

[1] St. Michael's Hospital has received funding from the Local Poverty Reduction Fund to conduct an **impact and process evaluation only,** and is expected to maintain an "arms-length" distance from the intervention.

be made to recruit young people facing additional identity-related challenges like newcomer and racialized youth, Indigenous youth, and youth who belong to the LGBTQ2S (lesbian, gay, bisexual, transgender, transsexual, queer, questioning, and 2-Spirit) community.

2. Evaluation Goal and Objectives

This impact and process evaluation aims to address critical gaps in our knowledge about transition-related supports by asking whether and how an identity capital intervention delivered outside the social service sector impacts the life-trajectories of formerly homeless young people. Particular attention will be paid to whether this intervention shows promise as an unconventional way to tackle poverty and improve social inclusion.

Evaluation Objectives

Specifically, this evaluation will investigate and disseminate important new knowledge on the following short-, medium-, and long-term outcomes (also see Appendix A – Program Logic Model):

1. The impact of the intervention on measures of **hope, community integration, social connectedness, and self-esteem.**
2. The effectiveness of the intervention to increase the number of young people in **education, employment, or training**.
3. Understanding any **practical challenges** encountered during the intervention implementation and how these challenges were resolved.
4. Understanding what young people found **most beneficial** about the intervention and their suggestions for how it could **be improved**.
5. The effectiveness of the intervention to decrease the number of young people who **return to homelessness**.
6. The effectiveness of the intervention to decrease the number of young people who **live below the poverty line.**

3. Evaluation Design and Methods

In order to maintain the arms-length relationship required of all third-party evaluators involved in Local Poverty Reduction Fund initiatives, the study investigators will not be directly involved in the design or implementation of the intervention; however, they will

work closely with dk Leadership and Covenant House Toronto to ensure the intervention design aligns with the proposed evaluation methods.

Participants will attend **two full-day workshops and four half-day group coaching sessions** over the course of six weeks (see Appendix B – Proposed dk Leadership Intervention). The workshops and coaching sessions will be supplemented by on-line (e.g., podcasts) and print (e.g., workbooks) material. The full-day workshops will focus on identity-enhancing topics such as self-understanding, creating a life of meaning and purpose, and strategic career development. The half-day group coaching sessions will focus on workshop debriefing and on exploring solutions to transition-related challenges faced by study participants. The workshops and coaching sessions will be held at the dk Leadership office in Toronto and led by certified psychologists from dk Leadership.

The evaluation will employ a **mixed method prospective cohort hybrid design** with an intervention and a delayed intervention comparison group. Immediately after the six-week intervention is offered to the first group (the intervention group [n = 15]), that group will be compared to the delayed intervention comparison group (n = 15) receiving standard shelter-based, transition-related supports such as assistance finding housing, connection to employment and/or education, etc. This will help prove that the outcomes measured immediately after the intervention is offered the first time are due to the intervention and not to other factors such as the passage of time. Participants in the **delayed intervention comparison group** will be offered the same six-week dk Leadership intervention approximately **three months after those in the intervention group have completed the intervention.** After that, the study will become a ‘single arm’ study (both groups will have received the intervention and the quantitative data will be ‘pooled’). Both groups will be followed for nine months after they have finished the intervention. The sample size was based on the maximum number of participants dk Leadership enrolls in their workshops (related to group dynamics).

Evaluation Rationale and Hypothesis

We believe an impact and process evaluation utilizing a mixed method prospective cohort design with an intervention and delayed intervention comparison group is the most appropriate because it:

1. Addresses the crucial need to understand and disseminate evidence regarding the impact *and* process of interventions targeting homeless youth – both will help assess and inform questions related to scalability.
2. Provides a more complete and synergistic utilization of data.
3. Balances scientific rigor with the ethical principle of fairness.

We hypothesize that, for the **primary quantitative outcome measures** of hope, community integration, social connectedness, and self-esteem:

1. We will observe significant improvements in the mean scores of the intervention group compared to the delayed intervention comparison group immediately post-intervention.
2. We will observe significant improvements in the mean scores of both groups (intervention and delayed intervention) immediately post-intervention.
3. These significant improvements will be sustained in both groups for at least three months post-intervention.

Participant Eligibility and Recruitment: Youth

Thirty young people aged 18 – 26 years who have left homelessness within the past three years will be collaboratively recruited by dk Leadership and our community partner Covenant House Toronto. For the purposes of this study, youth living in supportive housing contexts (e.g., transitional housing) will be considered to have left homelessness and will be eligible to participate in the study. As previously mentioned, a concerted effort will be made to recruit young people facing additional identity-related challenges like newcomer and racialized youth, Indigenous youth, and youth who belong to the LGBTQ2S community.

In addition to the above eligibility criteria above, study participants must:

- Be able to provide informed consent (see Appendix C – Participant Information and Consent Form)

- Be fluent in English
- Commit to attending two full-day workshops and four half-day group coaching sessions over the course of six weeks (missing more than one workshop and two coaching sessions will disqualify participants from the study)

Youth Worker Participants

Dk Leadership will be offering six spots (three in each six-week program) to youth workers from Covenant House Toronto. This decision was made collaboratively between dk Leadership and Covenant House Toronto. The aim is to offer the youth participants a 'familiar face' at the dk Leadership program and to build capacity for Covenant House staff.

Consent Process: Youth Participants

Free and informed consent will be obtained verbally and in writing from all evaluation participants. A concerted effort has been made to ensure the consent form is in plain language. Highlighted throughout the document is the fact that informed consent is an ongoing process and can be negotiated at any time.

The co-investigator will call each recruited youth on the telephone and arrange to meet them at a location most convenient for them (will likely be at or near Covenant House Toronto). Potential evaluation participants will be given a copy of the participant information and consent form to read. This document will also be reviewed verbally to ensure that those who have low literacy levels have been given the information required to give informed consent. During this process, the co-investigator will use her knowledge, skill and judgment as a nurse practitioner who works exclusively with street-involved and homeless youth to assess the capacity for the potential participant to provide informed consent. If it is determined that a youth is not able to consent, they will be informed of this when meeting with the co-investigator and they will be excluded from the evaluation.

Consent Process: Youth Workers

Youth workers who participate in the dk Leadership program will be invited by the co-investigator to participate in one focus group. This particular focus group will only be open to the youth workers and will take place immediately after each six-week program.

The co-investigator will reach out to the youth workers in-person or via e-mail during the last week of the six-week dk Leadership program. Potential focus group participants will be given a copy of the participant information and consent form to read (Appendix D). A concerted effort has been made to ensure the consent form is in plain language. Highlighted throughout the document is the fact that free and informed consent is an ongoing process and can be negotiated at any time. In addition, we have made clear that the decision to participate or not participate in the evaluation in no way impacts their employment at Covenant House Toronto.

4. Data Generation

This evaluation is a mixed-methods design with a strong qualitative component that draws on the expertise of the co-investigator (Dr. Naomi Thulien) in ethnographic methodology. We believe the qualitative data will facilitate an enhanced understanding of the quantitative data and provide a crucial (and underutilized) youth-informed perspective on poverty reduction and social inclusion.

Quantitative Measures

Quantitative data (see Appendix E – Quantitative Data Collection) for the **intervention group** will be collected at **five points** in time over the course of approximately 11 months: **baseline, immediately post-intervention (six weeks), and at three, six, and nine months post-intervention**. Quantitative data for the **delayed intervention group** will be collected at **six points** in time over the course of approximately 14 months: **baseline, six weeks, immediately post-intervention, and at three, six, and nine months post-intervention**.

At each data collection period, participants will be asked whether they are enrolled in **education, employment, or training**. They will also be asked about their **housing status**. In

addition, **four standardized measures** (see below) that have previously been utilized with homeless young people will be employed to assess the primary outcome variables.

Table 1. Quantitative Instruments

Instrument	Details
Beck Hopelessness Scale[27]	This 20-item scale measures motivation, expectations, and feelings about the future (internal consistency $\alpha > .93$).
Community Integration Scale[28]	This 11-item scale measures behavioral (e.g., participation in activities) and psychological (e.g., sense of belonging) aspects of community integration. This scale was used extensively in the Chez Soi/At Home Study but psychometric properties have yet to be reported.[28]
Social Connectedness Scale – Revised[29]	This 20-item scale measures belongingness – the degree to which people feel connected to others (internal consistency $\alpha > .92$).
Rosenberg Self-Esteem Scale[30]	This 10-item scale measures global self-worth (internal consistency α .77 – .88).

Qualitative Measures

Qualitative measures (see Appendix F – Qualitative Data Generation) are an important feature of this evaluation and will consist of: **1) participant observation and informal interviews, 2) semi-structured individual interviews, and 3) focus groups.**

Over the six-week intervention period, the co-investigator will attend the weekly meetings as an observer. She will do this with both the intervention and the delayed intervention group. During these meetings, the investigator will take field notes, documenting her perceptions about the way the workshops and group coaching sessions are being run, and how they are being 'taken up' by the young people in the intervention group. Additionally, the investigator will conduct informal interviews with the youth during the **six-week participant observation** period. These informal interviews will act as a form of data triangulation, allowing the researcher to confirm inferences made through participant observation.[31-32] The

informal interviews will be brief and conversational in nature and will be conducted at opportune moments during the workshops or group coaching sessions (e.g., during coffee or lunch breaks). Data generated during the informal interviews will be documented contemporaneously and incorporated into the field notes. The informal interviews will not be audio recorded.

All of the participants will be invited to attend **two focus groups,** which will be conducted **immediately post-intervention** and at **six months post-intervention**. Each focus group will contain approximately 8 – 10 youth. Participants in the intervention and delayed intervention will be in separate focus groups. Key informants (n = 8) will be selected (four from each group) for **semi-structured individual interviews**, which will take place at **three months post-intervention** and **nine months post-intervention**. The questions posed during the focus groups and semi-structured interviews will be guided by the evaluation objectives, but will be conversational and exploratory in nature with particular attention to identity capital, life-trajectories, and social inclusion. It is anticipated that the focus groups will last 60 – 90 minutes and the individual interviews 30 – 60 minutes. All will be conducted by the co-investigator at locations most convenient for the study participants, and will be audio recorded and transcribed verbatim.

Honorariums

All of the study participants will be paid an honorarium of $20 at each of the five data collection points (the quantitative data will be collected at the same time as the semi-structured interviews and focus groups). This amount was based on the co-investigator's previous experience with this demographic and after consulting with Covenant House Toronto.

5. Data Analysis

Quantitative Data

The frequencies, means and standard deviations will be calculated for all evaluation variables. To address the question about the extent to which the outcome variables change over time, we will conduct independent *t*-tests on the continuous variables (hope, community

integration, social connectedness, and self-esteem) and chi-square tests on the categorical variables (education, employment, training, and housing) to report on changes between: 1) baseline and immediately post-intervention; 2) baseline and three months post-intervention; 3) baseline and six months post-intervention; and 4) baseline and nine months post-intervention.[33-34] To address the question about whether the intervention group demonstrates a statistically significant difference in outcomes compared to no intervention, we will compare the outcome variables between the intervention group and the delayed intervention group six weeks after the baseline interviews (i.e., the intervention group will have just completed the dk Leadership program and the delayed intervention group will not have started it yet).

Qualitative Data

In keeping with the emergent, iterative nature of research using a qualitative design,[35-36] data analysis and interpretation will begin at the onset of the intervention. The handwritten field notes from the participant observation sessions will be typed into Microsoft Word by the co-investigator immediately after each biweekly meeting. The semi-structured individual interviews and focus groups will be audio recorded and transcribed verbatim. In order to conduct a more nuanced analysis of the data, the transcriptionist will be instructed to note short responses, uncooperative tones, and literal silence. [36-37] Prior to each meeting with the participants, the co-investigator will conduct a preliminary data analysis, reading the field notes or interview transcripts multiple times, separating the data into coded segments, making analytic memos beside large portions of the field notes or transcripts, identifying emerging themes (and comparing/contrasting these between participants), and complying new questions to ask the participants.[33,35] Participants will be asked for their perspectives on the emerging interpretations at each visit and these perspectives will play a key role in helping shape the data analysis and help ensure the trustworthiness of the data.[33,38] The web-based application Dedoose[39] will be utilized to assist with sorting and coding the qualitative data.

Quantitative and Qualitative Data Integration

In order to enhance the integration of the quantitative and qualitative data, the co-investigator will collect both forms of data at the focus groups and individual semi-structured interviews.

The quantitative measures will be collected first and then incorporated into the qualitative data generation. For example, if a participant indicates that they recently found employment or enrolled in job training, or if they score significantly high or low on the standardized measures, this can be explored further (as appropriate) during the focus group or individual interview. The research team will meet monthly to discuss the emerging analysis and to explore similarities or discrepancies between the quantitative and qualitative data.

6. Ethical Considerations

Benefits and Risks to Participants

Study participants may benefit from the opportunity to share with the co-investigator and with each other about whether and how an identity-building intervention delivered outside the social service sector impacts their life-trajectories. Additionally, participants may derive satisfaction from knowing that their contributions will help advance our understanding about how best to design interventions that help tackle poverty and improve social inclusion for young people transitioning out of homelessness.

The anticipated risks to participants are minimal. Participants will be assured that their participation or lack of participation will not affect their relationship with Covenant House Toronto. Participants may experience some emotional discomfort as a result of being asked about their transitions out of homelessness, which may be challenging. This will be minimized by reiterating to participants that participation is voluntary and that they can choose to withdraw from the study at any time. Additionally, the co-investigator will begin each semi-structured individual interview and focus group by emphasizing that a line of questioning may be stopped or redirected if the participant(s) is/are uncomfortable. If, during the semi-structured individual interviews or focus groups a study participant or the co-investigator feels that the participant could benefit from psychological support, the co-investigator will offer to connect them with a mental health counselor or psychiatrist at Covenant House Toronto (where the co-investigator practices) or with another appropriate mental health provider.

Privacy and Confidentiality

This mixed method evaluation will use multiple and varied data sources. This comprehensiveness is critical to the objectives of the evaluation, yet may increase invasion of participant privacy. This privacy concern will be clearly communicated to potential participants, as will the measures for protecting security and confidentiality, prior to consent. Any potential vulnerability of participants will be minimized since the evaluation findings will be de-identified and kept confidential, and because participants can choose not to answer any question(s) that they do not want or withdraw from the evaluation at any time.

All of the data collected will be kept in strict confidence. While participants' names will appear on the consent forms, pseudonyms (created by the participants) will be used in place of their real names on all documents related to data generation, including the audio recordings and transcripts from the individual interviews and focus groups. A key that links each participant name with a pseudonym will be stored on a separate encrypted USB flash drive and kept as a separate electronic file (see Appendix G – Linking Log). This separate encrypted USB flash drive will only be accessed by the co-investigator. Contact information for each participant will be entered using their pseudonym and stored on an encrypted USB flash drive only accessible to the co-investigator. The individual interviews and focus groups will be audio recorded using a password protected application within a password protected electronic device. The audio recordings from the individual interviews and focus groups will be deleted once the transcripts have been saved on an encrypted USB flash drive and entered into Dedoose (encrypted and password-protected).[39] Paper copies of the data (e.g., consent forms and standardized quantitative measures) will be stored in a locked filing cabinet at the Centre for Urban Health Solutions – an area only accessible to those with electronic key access. All paper and electronic files will be retained for a period of up to five years from study closure.

7. Significance

This partnership between dk Leadership and Covenant House Toronto represents a significant and innovative shift in the way we think about improving outcomes among marginalized young people. Rather than conceptualizing community-driven solutions to

poverty reduction as being the responsibility of the social service sector, we are witnessing the private sector ‘step-up’ and share in this responsibility. As clinician-researchers who work with the homeless, we are eager to evaluate this unconventional and promising approach to tackling poverty reduction and social inclusion. The findings from this mixed method impact and process evaluation will be an important contribution to our limited knowledge of effective evidence-based interventions for young people transitioning out of homelessness.

References

1. Gaetz, S., Dej, E., Richter, T., & Redman, M. (2016) *The state of homelessness in Canada 2016.* Toronto, ON: Canadian Observatory on Homelessness Press. Retrieved from http://www.homelesshub.ca/SOHC2016
2. Gaetz, S., & Redman, M. (2016). *Federal investment in youth homelessness: Comparing Canada and the United States and a proposal for reinvestment. Canadian observatory on homelessness policy brief.* Toronto, ON: The Homeless Hub Press. Retrieved from http://homelesshub.ca/sites/default/files/Policy_Brief.pdf
3. Covenant House Toronto. (2016). *Facts and stats*. Retrieved from http://www.covenanthousetoronto.ca/homeless-youth/Facts-and-Stats
4. Karabanow, J. (2008). Getting off the street: Exploring the process of young people's street exits. *American Behavioral Scientist, 51*(6)*,* 772-788. doi:10.1177/0002764207311987
5. Kidd, S.A., Frederick, T., Karabanow, J., Hughes, J., Naylor, T., & Barbic, S. (2016). A mixed methods study of recently homeless youth efforts to sustain housing and stability. *Child and Adolescent Social Work Journal*, *33*(3), 207-218. doi:10.1007/s10560-015-0424-2
6. Mayock, P., O'Sullivan, E., & Corr, M.L. (2011). Young people exiting homelessness: An exploration of process, meaning and definition. *Housing Studies, 26*(6), 803-826. doi:10.1080/02673037.2011.593131
7. Brueckner, M., Green, M., & Saggers, S. (2011). The trappings of home: Young homeless people's transitions towards independent living. *Housing Studies*, *26*(1), 1-16. doi:10.1080/02673037.2010.512751
8. Thulien, N.S., Gastaldo, D., Hwang, S.W., & McCay, E. (in press). The elusive goal of social integration: A critical examination of the socioeconomic and psychosocial consequences experienced by homeless young people who obtain housing. *Canadian Journal of Public Health, 109*(1)
9. Altena, A. M., Brilleslijper-Kater, S. N., & Wolf, J. R. (2010). Effective interventions for homeless youth: A systematic review. *American Journal of Preventive Medicine*, *38*(6), 637-645. doi: 10.1016/j.amepre.2010.02.017
10. Coren E., Hossain, R., Pardo, J.P., Veras, M., Chakraborty, K., Harris, H., Martin, A.J.

Interventions for promoting reintegration and reducing harmful behaviour and lifestyles in streetconnected children and young people. (2013). *Evidence-Based Child Health: A Cochrane Review Journal*, *8*(4), 1140-1272. doi: 10.1002/14651858.CD009823.pub2

11. Hwang, S.W., & Burns, T. (2014). Health interventions for people who are homeless. *The Lancet, 384*(9953), 1541-1547. Retrieved from http://www.thelancet.com
12. Gaetz, S. (2014). *Coming of age: Reimagining the response to youth homelessness in Canada.* Toronto, ON: The Canadian Homelessness Research Network Press. Retrieved from http://www.homelesshub.ca/comingofage
13. Karabanow, J., Carson, A., & Clement, P. (2010). *Leaving the streets: Stories of Canadian youth*. Halifax, NS: Fernwood Publishing.
14. Public Interest. (2009). *Changing patterns for street involved youth.* Toronto, ON: Author. Retrieved from http://www.worldvision.ca/Programs-and-Projects/Canadian Programs/Documents/ChangingPatternsForStreetInvolvedY outh.pdf
15. Milburn, N.G., Rice, E., Rotheram-Borus, M.J., Mallett, S., Rosenthal, D., Batterham, P., ... Duan, N. (2009). Adolescents exiting homelessness over two years: The risk amplification and abatement model. *Journal of Research on Adolescence, 19*(4), 762-785. doi:10.1111/j.1532-7795.2009.00610.x
16. Karabanow, J., Kidd, S., Frederick, T., & Hughes, J. (2016). Toward housing stability: Exiting homelessness as an emerging adult. *Journal of Sociology & Social Welfare, 43*(1), 121- 148. Retrieved from https://wmich.edu/socialworkjournal
17. Frederick, T., Chwalek, M., Hughes, J., Karabanow, J., & Kidd, S. (2014). How stable is stable? Defining and measuring housing stability. *Journal of Community Psychology, 42*(8), 964- 979. doi:10.1002/jcop.21665
18. Thulien, N.S. (2017). *Chronic precarity: A critical examination of homeless youth transitions to independent housing* (Doctoral dissertation). University of Toronto: Toronto, ON. Retrieved from https://tspace.library.utoronto.ca/bitstream/1807/78804/3/Thulien_Naomi_201706_PhD_thesis.pdf
19. Côté, J.E. (2016). *The identity capital model: A handbook of theory, methods, and findings.* Unpublished manuscript, Department of Sociology, The University of Western Ontario, London, Ontario, Canada. Retrieved from

https://www.researchgate.net/publication/305698905_The_Identity_Capital_Model_A_Handbook_Of_Theory_Methods_And_Findings

20. Oyserman, D., Bybee, D., & Terry, K. (2006). Possible selves and academic out-comes: How and when possible selves impel action. *Journal of Personality and Social Psychology*, *91*, 188-204. doi:10.1037/0022-3514.91.1.188
21. Lee, C., & Berrick, J.D. (2014). Experiences of youth who transition to adulthood out of care: Developing a theoretical framework. *Children and Youth Services Review*, *46*, 78-84. Retrieved from http://dx.doi.org/10.1016/j.childyouth.2014.08.005
22. Kidd, S., Frederick, T., Morales, S., Daley, M., & Vitopoulos, N. (2016, November). *The Toronto housing outreach program collaborative (HOP-C): Stabilizing housing for recently homeless youth*. Oral presentation at the Canadian Alliance to End Homelessness National Conference, Montreal, QC. Retrieved from http://conference.caeh.ca/wp-content/uploads/AWH5_The-Toronto-Housing-Outreach-Program-Collaborative-TFrederick-et-al.pdf
23. Oyserman, D., & Destin, M. (2010). Identity-based motivation: Implications for intervention. *The Counseling Psychologist*, *38*(7), 1001-1043. doi:10.1177/0011000010374775
24. Côté, J.E., & Schwartz, S.J. (2002). Comparing psychological and sociological approaches to identity: Identity status, identity capital, and the individualization process. *Journal of Adolescence*, *25*(6), 571-586. doi:10.1006/jado.2002.0511
25. dkLeadership. (2017). *Dream it. Do it.* Retrieved from http://www.dkleadership.org
26. Urbaniak, G.C., & Plous,S. (2013). Research Randomizer (Version 4.0) [Computer software]. Retrieved from https://www.randomizer.org
27. Beck, A. T., Weissman, A., Lester, D., & Trexler, L. (1974). The measurement of pessimism: The hopelessness scale. *Journal of Consulting and Clinical Psychology, 41*(6), 639–660. Retrieved from http://www.apa.org/pubs/journals/ccp/
28. Stergiopoulos, V., Gozdzik, A., O'Campo, P., Holtby, A., Jeyaratnam, J., & Tsemberis, S. (2014). Housing first: Exploring participants' early support needs. *BMC Health Services Research, 14*, 167. doi:10.1186/1472-6963-14-16
29. Lee, R. M., & Robbins S. B. (1995). Measuring belongingness: The social connectedness and the social assurance scales. *Journal of Counseling Psychology*, 42, 232-241

30. Rosenberg, M. (1965). Society and the adolescent self-image. Princeton, NJ: Princeton University Press.

31. Hammersley, M., & Atkinson, P. (2007). *Ethnography: Principles in Practice* (3rd ed.). London, UK: Routledge.

32. Kusenbach, M. (2003). Street phenomenology: The go-along as ethnographic research tool. *Ethnography, 4*(3), 455-485. Retrieved from http://eth.sagepub.com

33. Creswell, J.W. (2014). *Research design: Qualitative, quantitative, and mixed method approaches* (4th ed.). Thousand Oaks, CA: Sage.

34. Fletcher, R.H., Fletcher, S.W., & Fletcher, G.S. (2014). *Clinical epidemiology: The essentials* (5th ed.). Philadelphia, PA: Lippincott Williams & Wilkins.

35. Denzin, N.K., & Lincoln, Y.S. (2011). *The SAGE handbook of qualitative research*. Thousand Oaks, CA: SAGE Publications Inc.

36. Eakin, J.M., & Mykhalovskiy, E. (2003). Reframing the evaluation of qualitative health research: Reflections on a review of appraisal guidelines in the health sciences. *Journal of Evaluation in Clinical Practice*, *9*(2), 187-194. Retrieved from http://onlinelibrary.wiley.com/journal/10.1111/(ISSN)1365-2753

37. Kawabata, M., & Gastaldo, D. (2015). The less said, the better: Interpreting silence in qualitative research. *International Journal of Qualitative Research Methods, 14*(4), 1-9. doi:10.1177/1609406915618123

38. Loiselle, C.G., Profetto-McGrath, J., Polit, D.F., & Tatano Beck, C.T. (2004). *Canadian essentials of nursing research*. Philadelphia, PA: Lippincott Williams & Wilkins.

39. SocioCultural Research Consultants, LLC. (2016). Dedoose (Version 7.0.23) [web application]. Retrieved from http://www.dedoose.com

Appendix A

Program Logic Model

NAME OF PROJECT:
**The Identity Project: An Evaluation of an Identity Capital Intervention for Young People Transitioning Out of Homelessness**

PRIORITIES:
**To evaluate whether and how an identity capital intervention delivered outside the social service sector impacts the life-trajectories of formerly homeless young people**

INPUTS	OUTPUTS		OUTCOMES*		
	**Activities**	**Participants**	**Short-term (0-3 months)**	**Medium-term (3-9 months)**	**Long-term (9-12+ months)**
1. Academic, community-based, and private sector partnerships<br><br>2. Participation scholarships<br><br>3. Evaluation	1. Recruitment – 30 formerly homeless youth (15 - intervention group; 15 - wait-list comparison group)<br><br>2. Youth attend 2 workshops and 4 coaching sessions over 6 weeks – all designed to bolster identity capital<br><br>3. Collect quantitative data at baseline and 0, 3, 6, and 9 months post-intervention<br><br>4. Collect qualitative data at 0, 3, 6, and 9 months post-intervention	1. Covenant House and dk Leadership<br><br>2. Formerly homeless youth (within past 3 years)<br><br>3. C-UHS	1. Increase in motivation, goal setting, self-discipline, and organization (1st workshop + 2 coaching sessions)<br><br>2. Increase in self-awareness, confidence, ability to articulate career goals, and understanding how to achieve career goals (2nd workshop + 2 coaching sessions)	1. Increase in hope, community integration, social connectedness, and self-esteem<br><br>2. Increase in meaningful employment and/or engagement with school or training	1. Sustained improvement in medium-term outcomes<br><br>2. Decrease in youth living below the poverty line ($20,160/yr)<br><br>3. No return to homelessness<br><br>4. Improvement in overall sense of social inclusion<br><br>5. Youth-informed perspective (e.g., challenges/benefits) re: identity-building intervention

**ASSUMPTIONS**[17] (why identity matters)	**EXTERNAL FACTORS**** (identity-related inequities highlighted)
1. People act in identity-congruent ways<br>2. Formerly homeless youth have insufficient identity capital (sense of purpose and control, self-efficacy and self-esteem) to help them integrate into the mainstream<br>3. Formerly homeless youth find shelter-based supports stigmatizing (old identities)<br>4. Tangible assets (housing, education, and employment) **PLUS** intangible assets (identity capital) are needed to achieve meaningful social integration	1. Youth (13-25 years) comprise ~ 20% of the homeless population in Canada<br>2. Youth overrepresented in the Canadian homeless community include Indigenous youth, newcomer and racialized youth, and LGBTQ2S youth<br>3. 65% of Canadian homeless youth have not finished high school<br>4. 75% of Canadian homeless youth are not formally employed<br>5. 63% of Canadian homeless youth report childhood trauma and abuse

Short-term = during the 6-week intervention; ***medium-term*** = immediately to six months after the 6-week intervention; ***long-term*** = six to nine+ months after the 6-week intervention

**Stephen Gaetz, Bill O'Grady, Sean Kidd & Kaitlin Schwan. (2016). Without a Home: The National Youth Homelessness Survey. Toronto: Canadian Observatory on Homelessness Pres

Appendix B

Proposed dk Leadership Intervention

Time	Topics[2]
**Week One**	**Workshop #1: Emotional Intelligence and Confidence**<br>• *Understand how thoughts (positive or 'toxic') impact emotions and overall behavior (overall performance/decision-making)*<br>• *Understand how to identify and change 'toxic thoughts' and manage emotions*<br>• *Understand the impact of confidence on every part of life (e.g., relationships, goal-setting and risk-taking)*<br>• *Learn how to build confidence*<br>• *Understand how confidence is a core component of effective leadership*
**Week Two**	**Group Coaching #1**<br>• *Workshop review and debrief*<br>• *Further enhance skills around managing emotions and developing confidence*
**Week Three**	**Group Coaching #2**<br>• *Discuss barriers and facilitators to integrating key learnings from workshop and group coaching*<br>• *Reinforce and further develop goal-setting strategies and leadership skills*
**Week Four**	**Workshop #2: Motivation and Strategic Career Development**<br>• *Articulate dreams in six areas of life (relationships, recreation, career/school, health / fitness, spiritual & finance)*<br>• *Break down dreams into attainable goals (or mini-dreams) and create a 'vision board'*

[2] Workshops are full-day and group coaching sessions are half-day.

	• *Learn effective energy and time management techniques*<br>• *Take two 'personality tests' and learn what types of careers might align best with the results*<br>• *Learn what education and skills are needed for different careers and strategize about how to attain these*<br>• *Overview of "Top 50 Industry Experts" podcasts (available to participants after the workshop)*
**Week Five**	**Group Coaching #3**<br>• *Workshop review and debrief*<br>• *Review learning/themes from "Top 50 Industry Experts"*<br>• *Further enhance skills around energy and time management*
**Week Six**	**Group Coaching #4**<br>• *Discuss barriers and facilitators to integrating key learnings from workshops and group coaching*<br>• *Strategize specific next steps for goal attainment*<br>• *Overall program review and debrief*

Appendix C

Participant Information and Consent Form

St. Michael's
Inspired Care. Inspiring Science. | Centre for Urban Health Solutions

Study Title: The Identity Project: An Evaluation of an Identity Capital Intervention for Young People Transitioning Out of Homelessness

Principal Investigator: Stephen Hwang, MD, MPH, Director, Centre for Urban Health Solutions, Li Ka Shing Knowledge Institute of St. Michael's Hospital (416-864-5991)

Co-Investigator: Naomi Thulien, NP-PHC, PhD, Postdoctoral Fellow, Centre for Urban Health Solutions, Li Ka Shing Knowledge Institute of St. Michael's Hospital (647-460-0781)

Study Funder: Local Poverty Reduction Fund (Ontario Trillium Foundation)

You are being asked to consider participating in a research study. Before you give your consent, it is important that you read this form carefully so you understand what is being asked of you. Please let me know if there is anything on this form that you do not understand or anything that needs further explanation. You can change your mind about participating in this research at any time.

Conflict of Interest:

Dr. Naomi Thulien (the study co-investigator) is a nurse practitioner and medical director at Covenant House Toronto, the agency that helped recruit you for the dk Leadership training. Your participation in this evaluation (or decision to stop participating) in no way impacts your ability to receive healthcare services at Covenant House Toronto. Also, Dr. Thulien is listed on the dk Leadership as an "industry expert." This is related to an interview she did with them about being a nurse practitioner. She did not and does not receive money or any other kind of reimbursement from dk Leadership.

Why this research is being done:

We don't know enough about how best to support young people who have experienced homelessness once they are no longer homeless. From the limited research that has been done with young people who used to be homeless, we have learned that it can be really tough to move forward in life. Another thing we have learned is that how you feel about yourself (your sense of identity) and what

you *think* you can accomplish in life (also called identity capital) is really important, especially when life is challenging. Young people who used to be homeless have also told us that, while they want support, they would prefer to access these supports in locations not associated with homelessness, in part, because it reminds them of their old identities as homeless youth.

The purpose of this research is to evaluate the dk Leadership training that you will be attending. dk Leadership is an organization in Toronto that focuses on helping people understand themselves, develop leadership skills, and set career goals. The dk Leadership workshops and group coaching sessions are led by certified psychologists.

We want to understand whether and how this training impacts your identity and your ability to move forward in life. Some of the evaluation participants will take the dk Leadership training right away, while others will wait about three months after the first group has finished. This will allow us to compare the group that got the training right away with the group that waited. By having two groups take the training at different times, we can better understand if the changes we are seeing are due to the training or to something else.

Evaluation Design:

There will be approximately 30 youth participating in this evaluation. After you sign the consent form, a computer will help us place you randomly in one of two groups – like flipping a coin, this is called randomization. The first group (called the intervention group) will attend the six-week dk Leadership training right away and the second group (called the wait-list comparison group) will attend the dk Leadership training three months after the first group has finished. We will be following both groups for approximately **eleven months**.

Dr. Thulien will attend the dk Leadership training with the intervention group. During this training (immediately before or after a session, and at our coffee and/or lunch breaks), she will briefly ask questions about what participants think about the training. Over the one year that you are enrolled in this research, you may be invited to participate in **one or two focus groups** with approximately eight fellow training participants (should take about 60 – 90 minutes), and/or invited to participate in **one or two one-on-one interviews** (should take about 30 – 60 minutes). Also, every three months (five separate times over eleven months), we will ask all evaluation participants to fill out **five** short questionnaires (should take about 30 minutes).

What is required of you?

- A commitment to attend **two full-day** workshops and **four half-day** group coaching sessions at dk Leadership (2175 Danforth Avenue, Toronto, ON) over a six-week period
- A commitment to fill out **five short questionnaires** every three months for eleven months

NOTE: *You must participate in at least one full-day workshop and two half-day group coaching sessions to be involved in this evaluation.*

What happens when we meet?

This will depend on whether you are involved in the intervention or wait-list comparison group and whether you are participating in the focus groups and/or one-on-one interviews.

If you are in the intervention group, Dr. Thulien will have brief conversations with you while you are attending the dk Leadership workshops and group coaching sessions. After you are done talking, she will make notes about your conversation. These brief conversations will not be audio recorded. She will also take notes during the workshops and group coaching sessions.

Everyone involved in the evaluation (those in the intervention *and* wait-list comparison groups) will be asked to fill out five short questionnaires every three months for eleven months. If you are also participating in a focus group or one-on-one interview that day, you will fill out the questionnaires at the same time. We do our best to hold the focus groups and one-on-one interviews at times and locations most convenient for you.

The focus group sessions will be led by Dr. Thulien and these will be audio recorded. She will also be the one conducting the one-on-one interviews, and these will be audio recorded too. If, at any time during the one-on-one interviews, you feel uncomfortable with the questions being asked, or if you want the audio recording to stop, just let Dr. Thulien know and she will stop.

What sorts of questions will you ask me?

The five short **questionnaires** that you fill out every three months will ask about your housing situation, whether you are employed, your income, and if you are enrolled in upgrading your education (like attending college or an apprenticeship program). Also included are questions about

whether you feel hopeful, connected to the community and/or other people, and how you feel about yourself.

During the **focus group sessions** and **one-on-one interviews**, we will discuss the above questions in more detail. For example, if several people in the focus group sessions are not feeling very hopeful, we will explore this further. We will also discuss your thoughts about the dk Leadership training (e.g., what worked and what did not) and whether and how this training is helping you move forward in life.

Will I get reimbursed for my time?
Each time we meet you will receive $20.00.

What if I change my mind about participating?
You can change your mind about participating in the evaluation at any time and will not impact your care or relationship with the Covenant House or St. Michael's Hospital in any way. Even after the evaluation is over you can contact us if you change your mind about sharing some of the things we talked about. However, it is important to keep in mind that, after the final data analysis is completed, we will be unable to retract some of the information you have provided (e.g., your responses to the short questionnaires).

We will inform you as soon as possible if any information becomes available that may be relevant to you wanting to continue with this research.

NOTE: *If you are no longer able to continue in the evaluation, we will still use the information you provided unless you tell us not to, which you can do at any time.*

Potential Harm:
We do not anticipate that any harm will come to you because of your participation in this research. You may feel uncomfortable or find it stressful if we are asking too many questions, or asking questions that are really personal. Please let us know if this happens and we will stop or ask different questions. You do not have to talk about anything you are not comfortable with. Should the need arise, we can connect you with counselling services at Covenant House Toronto or at another organization.

Potential Benefit:

There is no direct benefit to you for participating in this research. You may find it helpful to participate in the dk Leadership training and to share what that experience was like for you. You may also find it helpful to share with us and/or with other participants about what it is like to move forward in life after experiencing homelessness.

Privacy and Confidentiality:

Anything you share will be kept in strict confidence. The only limit to this is if you tell us that you have a plan to hurt yourself or hurt other people, or if you tell us about someone under the age of 16 who is suffering abuse and/or neglect. If this happens, we are required by law to report this to the appropriate authorities.

Each research participant will create a pseudonym (fake name) that replaces their real name. This pseudonym will appear on all documents related to this research. In addition, whenever we talk about this research, we will only use your pseudonym, and never your real name. Any paper documents related to this research (like the questionnaires you fill out) will be stored in a secure location only accessible by the study team. All typed documentation related to this study will be stored on an encrypted USB memory key and password-protected computer. All participants in the focus group will be reminded to keep the discussion confidential; but the investigators cannot guarantee that your information or responses will not be shared by other participants.

Audio-recordings: The focus group sessions and one-on-one interviews will be audio-recorded. The audio-recordings will only be heard by Dr. Thulien and the transcriptionist. The transcriptionist will be required to sign a confidentiality agreement (i.e., they are not allowed to discuss details of the audio recordings with other people). The digital audio files will be stored on a password-protected computer. After the interviews are transcribed and reviewed for accuracy, the digital recording will be deleted. As previously mentioned, during the one-on-one interviews, you can tell us to stop audio recording at any time.

When will I learn about the results of the evaluation?

We would be happy to share the preliminary evaluation findings with you each time we meet to fill out the short questionnaires. In addition, those involved in focus groups and/or one-on-one interviews

will have the opportunity to discuss the emerging findings in more detail. If you have questions about the results of the evaluation at any time during or after the study, please contact us (contact information below) and we would be happy to discuss the results with you.

Who will you share the results of this evaluation with?

We will share the evaluation findings with the organization that funded this evaluation (Ontario Trillium Foundation) and well as Covenant House Toronto and dk Leadership. We also hope to share the results of this study with other social service providers (e.g., agencies working with street-involved youth), policy makers (e.g., politicians), university students, the general public, and at professional conferences. In addition, our goal is to publish the study findings in a few academic journals. Again – we will never use your real name.

Questions?

You can contact Dr. Naomi Thulien at: 647-460-0781 (24 hours) or ThulienNa@smh.ca. Alternatively, you can contact her Postdoctoral supervisor (Dr. Stephen Hwang) at: 416-864-5991or HWANGS@smh.ca.

This study has been reviewed by the Research and Ethics Board at St. Michael's Hospital. If you have any questions related to your rights as a study participant or about ethical issues related to this study, you can contact the Chair, Research Ethics at St. Michael's Hospital at: FreitagS@smh.ca or 416-864-6060 Ext. 2557

Study Title: The Identity Project: An Evaluation of an Identity Capital Intervention for Young People Transitioning Out of Homelessness

Participant

By signing this form, I confirm that:

- This research has been fully explained to me
- All of my questions have been answered
- I understand what is required of me
- I understand the potential harm and benefit of participating in this study
- I understand that I can withdraw from the research or change my mind about letting you use the information I have provided at anytime
- I understand that I do not have to answer any questions or meet in places I am not comfortable with
- I have carefully read each page of this consent form
- I have been given a copy of the consent form

______________________	______________________	__________
Name of Participant (print)	Signature	Date

Investigator

By signing this form, I confirm that:

- This research and its purpose has been fully explained to the participant named above
- All questions posed by the participant have been answered
- I have given the participant a copy of the consent form

______________________	______________________	__________
Name of Investigator (print)	Signature	Date

Appendix D

Participant Information and Consent Form: Youth Worker Staff

St. Michael's
Inspired Care. Inspiring Science. | Centre for Urban Health Solutions

Study Title: The Identity Project: An Evaluation of an Identity Capital Intervention for Young People Transitioning Out of Homelessness

Principal Investigator: Stephen Hwang, MD, MPH, Director, Centre for Urban Health Solutions, Li Ka Shing Knowledge Institute of St. Michael's Hospital (416-864-5991)

Co-Investigator: Naomi Thulien, NP-PHC, PhD, Postdoctoral Fellow, Centre for Urban Health Solutions, Li Ka Shing Knowledge Institute of St. Michael's Hospital (647-460-0781)

Study Funder: Local Poverty Reduction Fund (Ontario Trillium Foundation)

You are being asked to consider participating in a research study. Specifically, you are being asked to participate in **one focus group**. Before you give your consent, it is important that you read this form carefully, so you understand what is being asked of you. Please let me know if there is anything on this form that you do not understand or anything that needs further explanation. You can change your mind about participating at any time. The decision to participate or not participate in no way impacts your employment at Covenant House Toronto.

Conflict of Interest:

Dr. Naomi Thulien is listed on the DK Leadership as an "industry expert." This is related to an interview she did with them about being a nurse practitioner. She did not and does not receive money or any other kind of reimbursement from DK Leadership.

Why this research is being done:

We do not know enough about how best to support young people who have experienced homelessness once they are no longer homeless. From the limited research that has been done with young people who used to be homeless, we have learned that it can be really tough to move forward in life. Another thing we have learned is that how young people feel about themselves

(sense of identity) and what young people *think* they can accomplish in life (also called identity capital) is really important, especially when life is challenging. Young people who used to be homeless have also told us that, while they want support, they would prefer to access these supports in locations not associated with homelessness, in part, because it reminds them of their old identities as homeless youth.

The purpose of this research is to evaluate the six-week DK Leadership training program being offered to formerly homeless young people. We want to understand whether and how this training impacts the participants' identities and their ability to move forward in life.

Why this focus group is being conducted:
This focus group is being conducted with youth workers who have taken some or all of the six-week DK Leadership training program with the participants. Given your expertise with young people who have experienced homelessness, we are really interested to learn about your perspective of the program.

What is required of you?
A commitment to attend **one focus group** session. The focus group discussion will last 60 – 90 minutes.

What will happen at the focus group?
The focus group will be led by Dr. Thulien and will be audio recorded. We will discuss your general impression of the DK Leadership training sessions (e.g., what worked and what did not) and whether and how this training might help young people who have experienced homelessness move forward in life.

Will I get compensated for my time?
We will not be providing any financial compensation to you for attending; however, we encourage you to ask your employer to consider the attendance part of your work day (as it was when you attended the DK Leadership sessions(s)). We will provide refreshments.

What if I change my mind about participating?

You can change your mind about participating in the focus group at any time. Even after the focus group is over you can contact us if you change your mind about sharing some of the things we talked about. However, it is important to keep in mind that, after the final data analysis is completed, we will be unable to retract the information you have provided.

NOTE: *If you need to leave during the focus group, we will still use the information you provided unless you tell us not to, which you can do at any time.*

Potential Harm:

We do not anticipate that any harm will come to you because of your participation in this research. You may feel uncomfortable if Dr. Thulien asks too many questions or asks questions that seem too personal. Please let her know if this happens and she will stop or ask different questions. You do not have to talk about anything you are not comfortable with.

Potential Benefit:

There is no direct benefit to you for participating in this research. You may find it helpful to share and to discuss with colleagues the experience of participating in the DK Leadership session(s) alongside young people who have experienced homelessness.

Privacy and Confidentiality:

Anything you share will be kept in strict confidence. All participants in the focus group will be reminded to keep the discussion confidential; but the investigators cannot guarantee that your information or responses will not be shared by other participants.

Whenever we talk about this research, we will only use pseudonyms, and never real names. Any paper documents related to this research (like the transcript from the focus group) will be stored in a secure location only accessible by the study team. All typed documentation related to this study will be stored on an encrypted USB memory key and password-protected computer.

Audio-recording: The focus group session will be audio-recorded. The audio-recording will only be heard by Dr. Thulien and the transcriptionist. The transcriptionist will be required to sign a confidentiality agreement (i.e., they are not allowed to discuss details of the audio recording with other people). The digital audio file will be stored on a password-protected computer. After the focus group session has been transcribed and reviewed for accuracy, the digital recording will be deleted.

When will I learn about the results of this research?

Those involved in focus group session will have the opportunity to learn about and to discuss the preliminary findings of this research. If you have questions about the results of this research at any time, please contact us (contact information below) and we would be happy to discuss the results with you.

Who will you share the results of this research with?

We will share the findings with the organization that funded this evaluation (Ontario Trillium Foundation) and with DK Leadership. We also hope to share the results of this study with social service providers (e.g., agencies working with youth experiencing homelessness), policy makers, university students, the general public, and at professional conferences. In addition, our goal is to publish the study findings in a few academic journals. Again – we will never use your real name.

Questions?

You can contact Dr. Naomi Thulien at: 647-460-0781 (24 hours) or ThulienNa@smh.ca. Alternatively, you can contact her Postdoctoral supervisor (Dr. Stephen Hwang) at: 416-864-5991or HWANGS@smh.ca.

This study has been reviewed by the Research and Ethics Board at St. Michael's Hospital. If you have any questions related to your rights as a study participant or about ethical issues related to this study, you can contact the Chair, Research Ethics at St. Michael's Hospital at: FreitagS@smh.ca or 416-864-6060 Ext. 2557.

Study Title: The Identity Project: An Evaluation of an Identity Capital Intervention for Young People Transitioning Out of Homelessness

<u>Participant</u>

By signing this form, I confirm that:

- This research has been fully explained to me
- All of my questions have been answered
- I understand what is required of me
- I understand the potential harm and benefit of participating in this focus group
- I understand that I can withdraw from the focus group or change my mind about letting you use the information I have provided at anytime
- I understand that I do not have to answer any questions I am not comfortable with
- I have carefully read each page of this consent form
- I have been given a copy of the consent form

______________________________	______________________________	_______________
Name of Participant (print)	Signature	Date

<u>Investigator</u>

By signing this form, I confirm that:

- This research and its purpose has been fully explained to the participant named above
- All questions posed by the participant have been answered
- I have given the participant a copy of the consent form

______________________________	______________________________	_______________
Name of Investigator (print)	Signature	Date

Appendix E

Quantitative Data Collection

St. Michael's
Inspired Care.
Inspiring Science. | Centre for Urban Health Solutions

Baseline Demographic Questionnaire

Pseudonym: ___________________________ **Date: ________________________**

Please answer the questions below. <u>You do not have to answer any questions you do not feel comfortable with</u>.

1. What is your age? _________________
2. Do you self-identify as: Male □ Female □ Neither □
3. What is your race/ethnic background? ______________________________
4. Were you born in Canada? Yes □ No □

   If no:

   Where were you born? ________________________________

   When did you come to Canada? _____________________________

   What is your immigration status? _____________________________
5. How long have you lived away from your biological parents? ______________________
6. Has the Children's Aid Society been involved with you or your family? Yes □ No □
7. Have you ever lived in a foster or group home? Yes □ No □
8. Do you have any children? Yes □ No □ If yes, where do they live?

   ____
9. What is the last grade you completed? ____________________
10. How many times have you tried living on your own or with others after being homeless? _______

Thank you!!

St. Michael's
Inspired Care. Inspiring Science. | Centre for Urban Health Solutions

Quarterly Questionnaire

Pseudonym: ________________________

Date: ______________________

Time (circle): B, T1, T2, T3, T4

Please answer the questions below. <u>You do not have to answer any questions you do not feel comfortable with</u>.

1. Are you a student in secondary (e.g., high school) or post-secondary (e.g., college) institution? Yes □ No □

   If yes, what kind of school are you attending? ______________________

   If enrolled in post-secondary education, how are you paying for this?

   ____

2. Are you attending an employment training program? Yes □ No □

   If yes, what kind? ____

   If yes:

   a) Are you getting paid while you train? Yes □ No □
   b) If yes, how much? __________________________

3. Do you have a job? Yes □ No □

   If yes:

   a) What type of job? _______________________________
   b) How many hours do you work per week? ___________________
   c) How much money do you make each month? ________________

4. Are you making money outside of "regular/typical" employment? Yes □ No □

   If yes:

   a) Doing what? _______________________
   b) How much money do you make each month? ________________

5. Are you on OW or ODSP? Yes □ No
6. What is your current living situation? _____________________________________
7. How much is your rent each month? ______________________

Thank you!!

St. Michael's
Inspired Care. Inspiring Science. | Centre for Urban Health Solutions

Beck's Hopelessness Scale

Pseudonym: ______________________

Date: ______________________

Time (circle): B, T1, T2, T3, T4

Please circle the response that describes your attitude for the past week.

1. I look forward to the future with hope and enthusiasm.
   True/False
2. I might as well give up because there is nothing I can do to make things better for myself.
   True/False
3. When things are going badly, I am helped by knowing that they can't stay that way forever.
   True/False
4. I can't imagine what my life would be like in ten years.
   True/False
5. I have enough time to accomplish the things I most want to do.
   True/False
6. In the future, I expect to succeed in what concerns me most.
   True/False
7. My future seems dark to me.
   True/False
8. I happen to be particularly lucky and I expect to get more of the good things in life than the average person.
   True/False
9. I just don't get any breaks, and there's no reason to believe that I will in the future.
   True/False
10. My past experiences have prepared me well for my future.
    True/False
11. All I can see ahead of me is unpleasantness rather than pleasantness.
    True/False
12. I don't expect to get what I really want.

True/False

13. When I look ahead to the future I expect I will be happier than I am now.

    True/False

14. Things just won't work out the way I want them to.

    True/False

15. I have great faith in the future.

    True/False

16. I never get what I want, so it's foolish to want anything.

    True/False

17. It is very unlikely that I will get any real satisfaction in the future.

    True/False

18. The future seems vague and uncertain to me.

    True/False

19. I can look forward to more good times than bad times.

    True/False

20. There is no use in really trying to get something I want because I probably won't get it.

    True/False

TOTAL TRUE: __________ TOTAL FALSE: _____________

St. Michael's
Inspired Care. Inspiring Science. | Centre for Urban Health Solutions

Community Integration Scale

Pseudonym: ______________________________

Date: ______________________

Time (circle): B, T1, T2, T3, T4

Please circle your response to each question or statement.

Physical Integration

In the past month have you:

1. Attended a movie or concert?

   Yes No Don't remember

2. Participated in outside sports or recreation?

   Yes No Don't remember

3. Gone to meet people at a restaurant or coffee shop?

   Yes No Don't remember

4. Participated in a community event?

   Yes No Don't remember

5. Gone to a place of worship or participated in a spiritual ceremony?

   Yes No Don't remember

6. Participated in a volunteer activity?

   Yes No Don't remember

7. Gone to a library?

   Yes No Don't remember

TOTAL: Yes: _____ No: _____ Don't remember: ______

Psychological Integration

8. I know most of the people who live near me.

   1 - Strongly disagree 2 - Disagree 3 - Neither 4 - Agree 5 - Strongly agree

9. I interact with people who live near me.

   1 - Strongly disagree 2 - Disagree 3 - Neither 4 - Agree 5 - Strongly agree

10. I feel at home where I live.

    1 - Strongly disagree 2 - Disagree 3 - Neither 4 - Agree 5 - Strongly agree

11. I feel like I belong where I live.

    1 - Strongly disagree 2 - Disagree 3 - Neither 4 - Agree 5 - Strongly agree

TOTAL SCORE: _________

St. Michael's
Inspired Care. Inspiring Science. | Centre for Urban Health Solutions

Social Connectedness Scale – Revised

Pseudonym: ____________________

Date: ____________________ **Time (circle): B, T1, T2, T3, T4**

Please circle your response to each statement.

1. I feel comfortable in the presence of strangers.

1-Strongly disagree 2-Disagree 3-Mildly disagree 4-Mildly agree 5-Agree 6-Strongly agree

2. I am in tune with the world.

1-Strongly disagree 2-Disagree 3-Mildly disagree 4-Mildly agree 5-Agree 6-Strongly agree

3. Even among my friends, there is no sense of brother/sisterhood.

6-Strongly disagree 5-Disagree 4-Mildly disagree 3-Mildly agree 2-Agree 1-Strongly agree

4. I fit in well in new situations.

1-Strongly disagree 2-Disagree 3-Mildly disagree 4-Mildly agree 5-Agree 6 -Strongly agree

5. I feel close to people.

1-Strongly disagree 2-Disagree 3-Mildly disagree 4-Mildly agree 5-Agree 6-Strongly agree

6. I feel disconnected from the world around me.

6-Strongly disagree 5-Disagree 4-Mildly disagree 3-Mildly agree 2-Agree 1-Strongly agree

7. Even around people I know, I don't feel that I really belong.

6-Strongly disagree 5-Disagree 4-Mildly disagree 3-Mildly agree 2-Agree 1-Strongly agree

8. I see people as friendly and approachable.

1-Strongly disagree 2-Disagree 3-Mildly disagree 4-Mildly agree 5-Agree 6-Strongly agree

9. I feel like an outsider.

6-Strongly disagree 5-Disagree 4-Mildly disagree 3-Mildly agree 2-Agree 1-Strongly agree

10. I feel understood by the people I know.

1-Strongly disagree 2-Disagree 3-Mildly disagree 4-Mildly agree 5-Agree 6-Strongly agree

11. I feel distant from people.

6-Strongly disagree 5-Disagree 4-Mildly disagree 3-Mildly agree 2-Agree 1-Strongly agree

12. I am able to relate to my peers.

1-Strongly disagree 2-Disagree 3-Mildly disagree 4-Mildly agree 5-Agree 6-Strongly agree

13. I have little sense of togetherness with my peers.

6-Strongly disagree 5-Disagree 4-Mildly disagree 3-Mildly agree 2-Agree 1-Strongly agree

14. I find myself actively involved in people's lives.

1-Strongly disagree 2-Disagree 3-Mildly disagree 4-Mildly agree 5-Agree 6-Strongly agree

15. I catch myself losing a sense of connectedness to society.

6-Strongly disagree 5-Disagree 4-Mildly disagree 3-Mildly agree 2-Agree 1-Strongly agree

16. I am able to connect with other people.

1-Strongly disagree 2-Disagree 3-Mildly disagree 4-Mildly agree 5-Agree 6-Strongly agree

17. I see myself as a loner.

6-Strongly disagree 5-Disagree 4-Mildly disagree 3-Mildly agree 2-Agree 1-Strongly agree

18. I don't feel related to most people.

6-Strongly disagree 5-Disagree 4-Mildly disagree 3-Mildly agree 2-Agree 1-Strongly agree

19. My friends feel like family.

1-Strongly disagree 2-Disagree 3-Mildly disagree 4-Mildly agree 5-Agree 6-Strongly agree

20. I don't feel I participate with anyone or any group.

6-Strongly disagree 5-Disagree 4-Mildly disagree 3-Mildly agree 2-Agree 1-Strongly agree

TOTAL SCORE: ____________

St. Michael's
Inspired Care. Inspiring Science. | Centre for Urban Health Solutions

Rosenberg Self-Esteem Scale **Pseudonym: ______________________________**

Date: ____________________ **Time (circle): B, T1, T2, T3, T4**

Please circle your response to each statement.

1. On the whole, I am satisfied with myself.
   0 - Strongly disagree   1 - Disagree   2 - Agree   3 - Strongly agree
2. At times, I think I am no good at all.
   3 - Strongly disagree   2 - Disagree   1 - Agree   0 - Strongly agree
3. I feel that I have a number of good qualities.
   0 - Strongly disagree   1 - Disagree   2 - Agree   3 - Strongly agree
4. I am able to do things as well as most other people.
   0 - Strongly disagree   1 - Disagree   2 - Agree   3 - Strongly agree
5. I feel I do not have much to be proud of.
   3 - Strongly disagree   2 - Disagree   1 - Agree   0 - Strongly agree
6. I certainly feel useless at times.
   3 - Strongly disagree   2 - Disagree   1 - Agree   0 - Strongly agree
7. I fell that I'm a person of worth, at least on an equal plane with others.
   0 - Strongly disagree   1 - Disagree   2 - Agree   3 - Strongly agree
8. I wish I could have more respect for myself.
   3 - Strongly disagree   2 - Disagree   1 - Agree   0 - Strongly agree
9. All in all, I am inclined to feel that I am a failure.
   3 - Strongly disagree   2 - Disagree   1 - Agree   0 - Strongly agree
10. I take a positive attitude toward myself.
    0 - Strongly disagree   1 - Disagree   2 - Agree   3 - Strongly agree

TOTAL SCORE: ________

Appendix F

Qualitative Data Generation

St. Michael's
Inspired Care. Inspiring Science. | Centre for Urban Health Solutions

Initial Interview Guide: Individual Interviews and Focus Groups

1. *Overall sense of intervention*
    a) Can you describe, in general, what you thought of the dk Leadership program?
    b) Was it different than you were expecting? If so, how?
    c) Can you give me an example of something you learned that you did not know before?
    d) What did you think of the location?
    e) Do you see yourself and/or the future differently after attending the program? If so, can you give me an example?

2. *Practical challenges*
    a) Can you help me understand some practical challenges of attending the program?
    b) What advice do you have for dk Leadership and/or for other program participants to help overcome these challenges?

3. *Benefits of intervention*
    a) What was the best thing about the dk Leadership program?
    b) Can you describe the qualities you think are most important in a program leader?
    c) Can you give me an example of how this program has helped you move forward, or think about moving forward in life?

4. *Suggested improvements to intervention*
    a) If you were running the same dk Leadership program that you attended, how would you change it?

b) If you had a magic wand, what would be an ideal program for young people who have experienced homelessness? Where would it be located? How long would it last?

5. *Social inclusion (financial, social, and identity capital)*
    a) When you spend time around people who have not experienced homelessness, how do you feel?
    b) What do you hope your life look like in five years? Ten years?
    c) How confident are you that you can achieve what you hope for your life? Did attending the dk Leadership program have any impact on this confidence?
    d) Can you help me understand some of the things you think might hold you back from achieving what you hope for in life?
    e) Can you name me someone you admire? Why?

These questions will be used to guide the initial individual interviews and focus groups. They are based on the overall evaluation objective of understanding whether and how an identity capital intervention delivered outside the social service sector impacts the life-trajectories of formerly homeless young people. It is expected that these questions will evolve over time.

Appendix G

Linking Log

St. Michael's
Inspired Care. Inspiring Science. | Centre for Urban Health Solutions

The Identity Project: Master Linking Log

Name (last, first)	DOB: (year/month/date)	Pseudonym
1.		
2.		
3.		
4.		
5.		
6.		
7.		
8.		
9.		
10.		
11.		
12.		
13.		
14.		
15.		
16.		
17.		
18.		
19.		
20.		
21.		
22.		
23.		
24.		
25.		
26.		
27.		
28.		
29.		
30.		
